# Multilevel structural evaluation of signed directed social networks based on balance theory

**DOI:** 10.1038/s41598-020-71838-6

**Published:** 2020-09-17

**Authors:** Samin Aref, Ly Dinh, Rezvaneh Rezapour, Jana Diesner

**Affiliations:** 1 Laboratory of Digital and Computational Demography, Max Planck Institute for Demographic Research, 18057 Rostock, Germany; 2 School of Information Sciences, University of Illinois at Urbana-Champaign, Champaign, USA

**Keywords:** Applied mathematics, Computational science, Information theory and computation, Computer science, Applied physics

## Abstract

Balance theory explains how network structural configurations relate to tension in social systems, which are commonly modeled as static undirected signed graphs. We expand this modeling approach by incorporating directionality of edges and considering three levels of analysis for balance assessment: triads, subgroups, and the whole network. For triad-level balance, we develop a new measure by utilizing semicycles that satisfy the condition of transitivity. For subgroup-level balance, we propose measures of cohesiveness (intra-group solidarity) and divisiveness (inter-group antagonism) to capture balance within and among subgroups. For network-level balance, we re-purpose the normalized line index to incorporate directionality and assess balance based on the proportion of edges whose position suits balance. Through comprehensive computational analyses, we quantify, analyze, and compare patterns of social structure in triads, subgroups, and the whole network across a range of social settings. We then apply our multilevel framework to examine balance in temporal and multilayer networks to demonstrates the generalizability of our approach. In most cases, we find relatively high balance across the three levels; providing another confirmation of balance theory. We also deliver empirical evidence for the argument that balance at different levels is not the same social phenomenon measured at different scales, but represents different properties (triadic balance, internal cohesion and external division of subgroups, and overall network polarization), and should therefore be evaluated independently from one another. We propose a comprehensive yet parsimonious approach to address this need.

## Introduction

Social entities can establish different types of relationships, such as personal or professional ones. These relationships might be reciprocal or not, and may have a dual and opposite nature, such as friendship versus enmity, and trust versus distrust. Over time and across types of relationships, ties may change. Signed directed graphs can be used to model such complex relationships as networks in which ties have two properties: a sign (positive or negative) and directionality. Additionally, the type and time of relationships can be modeled with layering and temporal edge attributes, respectively. Analyzing the resulting data allows for exploring the structure and dynamics of relationships among social entities, incorporating observations based on social science theories, and putting existing theories to empirical test, among other use cases.

One existing theory is structural balance^1,2^, which has been widely used to explain local-level social dynamics that emerge within and between triads, potentially causing a ripple throughout a network, leading to network-wide effects. With its root in social and cognitive psychology^1^, balance theory explains how different configurations of positive and negative relationships between pairs of nodes may impact the amount of tension in a closed triad (three nodes with an edge between every pair of nodes). This tension would be absent if the triad has an even number (0 or 2) of negative edges^2^. Applying this premise to real-world situations, the following four adages scope out the balanced configurations at the triadic level based on edge signs: *my friend’s friend is my friend* $(+++)$*; my friend’s enemy is my enemy* $(+--)$*; my enemy’s friend is my enemy* $(-+-)$*; and my enemy’s enemy is my friend* $(--+)$. The measurement of balance has later been expanded from the triads level (micro-level) to the subgroup level (meso-level) by partitioning nodes into two groups or “plus-sets”^3,4^, such that the number of positive edges between groups and negative edges within groups is decreased to some extent. The measurement of balance could also be expanded to the network level (macro-level) using the *line index of balance*^5–9^, which equals the minimum number of positive edges between groups and negative edges within groups across all possible ways to partition the nodes into two groups.

Looking at the prior body of research on balance, we conclude that for a more comprehensive analysis, balance should be assessed at multiple levels of the network, namely the micro-, meso-, and macro-level. We propose a new methodological framework to “link micro and macro levels”^10^ for analyzing signed social networks.

Structural balance has primarily been studied for undirected signed networks^8,11–13^ as opposed to directed signed networks^14–16^, although directed signed network data have been available since the early days of balance theory^17^. Using undirected network models for balance assessment could be justified when the modeled relationships are truly undirected, such as collaborations^18^, or inherently reciprocal, such as bi-lateral alliances^9^. However, many real-world relationships are intrinsically directed, such as social preferences^17,19^, and not necessarily reciprocated, such as a friendship^20^. Therefore, disregarding directionality^21^ when it does apply can jeopardize the validity of network measures^22^, including balance assessment^12,21^.

Several recent studies have implemented different methods and measurements to bridge the gap between balance theory and its evaluation on empirical data^8,23–25^. Empirical findings that support the theory seem to be mixed at the first glance. In the context of international relations, Maoz et al. ^23^ compared the probability of a negative edge between nodes sharing a common enemy to that of a random dyad. They observed the probability of a negative edge to be higher than they had expected, and considered this unexpected finding as an empirical evidence that contradicts balance theory. Later, Lerner^25^ explained the same contradictory observation to be an artifact of the method used by Maoz et al.^23^: arguing that by conditioning the probability on the presence of an edge, the empirical observations would become indicative of strong support for balance theory. Similarly, Estrada and Benzi proposed a measure of balance based on closed walks, and observed that balance in real signed networks is particularly low^24^, which also suggests a contradiction to balance theory. Later, Aref and Wilson^8^ showed that such observed low balance is an artifact of the walk-based method used by Estrada and Benzi^24^, which consistently shows low balance for real networks, even in cases where all alternative measures show high balance and provide strong evidence in support of balance theory. These are only a few examples for mixed observations on the applicability of balance theory, with the contradictions being explainable by the use of different methods for evaluating balance. This status quo shows that further methodological work is needed to bridge the gap between balance theory and empirical evaluations of balance.

Existing methods in network analysis, such as multilevel modeling^26^, relational event models (REMs)^27^, and exponential random graph models (ERGMs)^28^ have been used to examine balance dynamics at the micro- and macro-level. Kleinnijenhuis and de Nooy^26^ constructed a multilevel model to examine the extent to which a party’s position on an issue is influenced by the positions of their allies and opponents, and found that both micro-level balance and transitivity impact a party’s position towards an issue. Lerner and Lomi^27^ constructed an REM to examine micro- and macro-level patterns of positive and negative ties between Wikipedia editors and found that balance at the micro-level gave rise to a polarized macro-level structure, where events of the same sign between two editors were likely to occur again. Wang et al.^28^ conducted a series of experiments using ERGM to determine the extent to which triadic closure effects, along with other parameters, explain the structural characteristics of a network at the micro-, meso-, and macro-level. Their findings demonstrate structural dependencies between the three levels of analysis, where a change in one level may impact the configuration at another level. We build upon and extend this prior line of work by examining multiple levels of analysis with respect to structural balance.

This study makes two primary contributions to address the issues outlined above. First, we propose a multilevel evaluation framework that consists of balance measurement at three fundamental levels of analysis, namely at the (1) triad (micro), (2) subgroup (meso), and (3) network (macro) levels. Our definition of multilevel evaluation differs from prior literature on multilevel analysis or multilevel models^26^ in that our proposed framework does not deal with a statistical model with parameters varying in more than one level; neither do we consider the nested nature of the three levels of analysis^28,29^. Instead, we evaluate the three given levels of a network structure separately, and draw inferences from the results about the extent to which balance applies at each of the considered network levels. We then determine if measurements at different levels lead to different conclusions about balance in one and the same network. Second, to analyze balance, we consider both tie direction and sign. At the micro-level, we examine the balance of semicycles embedded within each triad. At the meso-level, we derive measures of cohesiveness and divisiveness to capture balance within and between subgroups. At the macro-level, we leverage a normalized version of the line index of balance^5^ (also known as the normalized frustration index^8,9^) to measure partial balance of a whole network.

We analyze 11 real-world networks to determine how balance manifests in different social settings (e.g., friendship among students, relationships among philosophers), in some cases with additional network properties (temporality, layering). Temporality is the multiplicity of snapshots of the same network over a period of time. The number of snapshots can be either one (static network), or more than one (temporal network). Layering is the number of different signed relationship types within the same network, which can either be one (single-layer network), or multiple (multilayer network). We divide our datasets into three categories with respect to the following dimensions: (a) static single-layer (static for brevity), (b) temporal (multiple snapshots), and (c) multilayer (multiple layers). We aim to answer the following research questions, which are methodological in nature:

- RQ1: How can we measure the balance of a static signed directed network at the micro-, meso-, and macro-level?
- RQ2: What insights do we gain from a multilevel evaluation of balance in a static signed directed network?
- RQ3: How is a multilevel evaluation of balance applied to a temporal or multilayer signed directed network?

Research questions 1 and 2 aim to identify a methodology for understanding balance as a potentially multi-faceted concept. Such a general method needs to be applicable to a wide range of networks that differ in social settings and structural properties. The third research question examines the applicability of a multilevel balance analysis to temporal and multilayer networks. In essence, our work identifies the extent to which networks are balanced depending on the level of analysis, thereby bringing together and building upon insights from previous studies that focus on a single level of analysis for one or more networks.

Our methodological contribution allows us to reflect on the substantive question about the extent to which social relations show consistency with balance theory^1,2,8,30^.

### Our contributions

- In this paper, we develop, operationalize, and test a novel measure for micro-level balance in signed directed networks. This measure accounts for edge directionality and brings together theoretical work on transitivity and balance theory.
- We apply balance theory to the meso-level of networks, and provide two new measures for the evaluation of balance that quantify the concepts of cohesiveness and divisiveness of subgroups.
- We re-purpose the frustration index, expand it to incorporate directionality, and develop an optimization model for computing the frustration index of directed signed networks.
- We propose a methodological framework with broad applicability to structural network analysis that allows for considering three levels of analysis to improve the comprehensiveness of evaluating directed signed networks.
- Our analysis of empirical network data demonstrates the relevance and suitability of our proposed framework not only for static networks, but also for directed signed network data with additional features (temporal and multilayer).

## Data description

One key purpose of this study is to present a single, general methodology for analyzing signed directed networks. Therefore, we demonstrate the application of our proposed method by analyzing 11 networks that contain signed directed ties and represent distinct social contexts. Table 1 provides details and descriptive information for each dataset considered herein (for further details see the Supplementary Information). We analyze eight static networks of different size, ranging from dozens to tens of thousands of edges. An example of a static network is *Reddit*, which denotes positive or negative sentiment (edge sign) of content shared between online accounts, and was captured at one point in time. Furthermore, we study two temporal networks, namely *Sampson’s monastery affect* data^31^ collected over three time periods (T2, T3, T4), and *Newcomb’s fraternity network*^19^, which entails 15 snapshots. Our version of the Sampson’s network data contain one type or layer of relationship and two possible values (positive or negative) of edges among 18 monks. Newcomb’s fraternity network contains one layer of relationship with two possible values (positive or negative) of edges among 17 fraternity brothers living in a shared residence^19^. Finally, we examine one multilayer social network, *Collins’ philosophers network*^32^, which contains one snapshot of two types of signed relationships (master-pupil and acquaintanceship) between philosophers from different schools of thoughts.

All data used in this study are publicly available under a CC BY 4.0 license in a FigShare data repository^33^.

**Table 1:** List of directed signed networks datasets used in our study.

Dataset	*n* (# of Nodes)	*m* (# of Edges) $(m^+,m^-)$	Type of network		Description
			# of Snapshots	# of Layers	
Reddit^34^	18,313	120,792 (111,891, 8,901)	1	1	Represents connections between users of two subreddits from Jan 2014 to April 2017. Collected online
Wikipedia election^35^	7,118	103,675 (81,318, 22,357)	1	1	Contains approval and disapproval votes for electing admins in Wikipedia from 2003 to 2013. Collected online
Bitcoin OTC^36^	5,881	35,592 (32,029, 3,563)	1	1	Represents the record of reputation/trust of users on a Bitcoin trading platform. Collected online
Bitcoin Alpha^36^	3,783	24,186 (22,650, 1,536)	1	1	Represents the record of reputation/trust of users on a Bitcoin trading platform. Collected online
Highland tribes^37^	16	116 (58, 58)	1	1	Represents alliance structure among three tribal groups. Collected offline
College preferences House A^17^	21	94 (51, 43)	1	1	Preference rankings of a group of girls in an Eastern college. Collected offline
College preferences House B^17^	17	83 (41, 42)	1	1	Preference rankings of a group of girls in an Eastern college. Collected offline
College preferences House C^17^	20	81 (41, 40)	1	1	Preference rankings of a group of girls in an Eastern college. Collected offline
Sampson’s affect^31^	18	T2: 104 (55, 49) T3: 105 (57, 48) T4: 103 (56, 47)	3	1	Represents social relationships among 18 monk-novitiates over 3 time periods. Collected offline
Newcomb’s fraternity^19^	17	All snapshots: 102 (51, 51)	15	1	Preference rankings of 17 boys in a pseudo-dormitory over 13 weeks. Collected offline
Philosophers network^32^	855	2,010 (1,736, 274)	1	2	Acquaintanceship and master-pupil relationships among philosophers recorded by Collins in^32^. Collected offline

## Notations and basic definitions

We denote a directed signed graph as $G = (V,E,\sigma )$, where *V* and *E* are sets of vertices and directed edges, respectively, and $\sigma$ is the sign function that maps edges to $\{-1,+1\}$. A signed digraph *G* contains $|V|=n$ nodes and $|E|=m$ directed edges. The set *E* of directed edges contains $m^-$ negative edges and $m^+$ positive edges.

**Figure 1:**
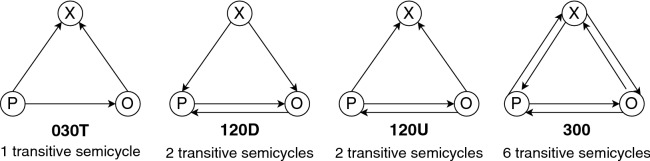
Triads in the triad census^41^ with transitive semicycles. Signs of edges (not shown in the figure) can either be positive or negative. Triad types are labeled based on the number of mutual (first digit), asymmetric (second digit), and null (third digit) dyads, and an additional letter for direction (T: transitive, D: down, U: up). See^41^ for more details about nomenclature for the triad census.

A *triad* in *G* is a set of three nodes with at least one directed edge between each two of them (could be in either direction). Figure 1 shows 4 triads. Given a triad, if there are 3 edges incident on its nodes such that for every pair of nodes there is one edge, then those three edges form a *semicycle*. A triad has at least one semicycle but it could also have multiple semicycles. In Fig. 1, the leftmost triad has one semicycle while the rightmost triad has eight semicycles. If the binary relation ${\mathcal {R}}$ that defines edges $A{\mathcal {R}}B \leftrightarrow (A,B)\in E$ is transitive over the set of a semicycle’s edges (i.e. $A{\mathcal {R}}B \ \& \ B{\mathcal {R}}C \rightarrow A{\mathcal {R}}C$), the semicycle is called a *transitive semicycle*. A transitive semicycle is balanced (unbalanced) if and only if the product of the signs on its edges is positive (negative). A signed digraph is balanced if and only if its set of nodes can be partitioned into two groups such that all positive edges are within each group and all negative edges are between the groups.

## Multilevel evaluation of balance

In this section, we discuss our proposed multilevel evaluation framework which involves measuring balance at the micro-, meso-, and macro-level.

### Measuring balance at the micro-level

To evaluate balance in a signed network, the most common method is to quantify balance per triad^2,14,38,39^. This step is usually followed by adding up and comparing frequencies or ratios of balanced versus unbalanced triads, with the implicit assumption being that this aggregation represents a network’s overall balance. The majority of studies do not consider edge directionality when calculating triadic balance. In real-world social networks with positive and negative relationships, ties are not necessarily reciprocated. For instance, A might perceive B as a friend, but B is neutral towards A, which can be formulated as $(A,B) \in E^+, (B,A) \notin E$ using a signed digraph notation. Another example would be A trusting B but B distrusting A, which can be formulated as $(A,B) \in E^+, (B,A) \in E^-$. Undirected signed networks are incapable of modeling such basic cases, leading to the exclusion of these situations from network models^21^ or the disregard of all unreciprocated edges for analysis^12,40^. This fundamental flaw is resolved by using signed digraphs, which results in a more flexible and comprehensive network model. Addressing this problem requires the consideration of edge directionality for measuring balance. Our unit of analysis for the micro-level evaluation of balance is a transitive semicycle. We only evaluate triads in which most semicycles are transitive (which we refer to as transitive triads). Four types of triads (as in the triad census^41^: ‘030T’, ‘120D’, ‘120U’, and ‘300’) are transitive (illustrated in Fig. 1). For triad ‘300’, we only consider its six transitive semicycles, and disregard its two cyclic semicycles. For triads ‘030T’, ‘120D’, and ‘120U’, we consider all their semicycles because they are all transitive. A transitive triad is balanced if all of its transitive semicycles are balanced. For our analysis, we use the *NetworkX* library in *Python* to assess the balance of triads, and obtain *T*(*G*) as the fraction of balanced transitive triads over all transitive triads.

Evaluating balance solely at the micro-level is common practice, but rests on the assumption that aggregating triad-level balance is sufficient to determine network-level balance. Also, measuring balance at the triad level does not consider how configurations within triads influence neighboring nodes and edges as well as broader areas of the network. Based on prior literature, there are structural configurations beyond the triad, such as longer cycles, that contribute to balance of a network or lack thereof^3,5,8,9,24,42^. These findings show that simply aggregating balance scores from the micro-level might not capture other structural features such as density. To mitigate the limitations resulting from a single-level evaluation, we propose and apply complementary methods to evaluate meso- and macro-level balance as parts of a comprehensive multilevel evaluation framework.

### Measuring balance at the meso-level

To evaluate meso-level balance, vertices of a network can be partitioned into two mutually antagonist but internally solidary subgroups^7,43–45^. We refer to solidarity within subgroups as cohesiveness, and to antagonism between subgroups as divisiveness within a network. An internally solidary subgroup means that there are only positive edges within a subgroup. Two internally solidary subgroups are mutually antagonistic when they are connected by only negative edges. This approach returns the minimum number of negative edges within subgroups and positive edges between subgroups across all possible ways of partitioning nodes into two subsets. To demonstrate our approach for quantifying these two new measures, we start with an extreme yet illustrative example: a balanced network that contains both positive and negative edges has an extreme amount of cohesiveness (because all edges within its two subgroups are positive) and an extreme amount of divisiveness (because all edges between its two subgroups are negative). We quantify cohesiveness and divisiveness through the deviation from this extreme case.

Using a signed digraph $G=(V,E,\sigma )$ as input, the set of vertices, *V*, can be partitioned based on $P=\{X,V{\setminus } X\}$ into the two subgroups *X* and $V{\setminus } X$. Given partition $P=\{X,V{\setminus } X\}$, edges that cross the subgroups are *external* edges that belong to $E^e_P=\{(i,j)\in E |i\in X, j\notin X \ \text {or} \ i\notin X, j\in X\}$. Edges that do not cross the subgroups are *internal* edges that belong to $E^i_P=\{(i,j)\in E |i,j\in X \ \text {or} \ i,j\notin X\}$. We measure cohesiveness (divisiveness) of a partition *P* by only looking at the signs of its internal (external) edges. We quantify the cohesiveness of a given partition *P* using the fraction of its positive internal edges to all internal edges $C(P)={| E^i_P \cap E^+|}/{|E^i_P|}$. Similarly, we quantify the divisiveness of partition *P* as the fraction of its negative external edges to all external edges $D(P)={| E^e_P \cap E^-|}/{|E^e_P|}$. We compute cohesiveness and divisiveness using $P^*$, which is the best fitting bi-partition of nodes, as explained further in the next subsection. This bi-partition is also connected to our proposed macro-level analysis. Our proposed measures of cohesiveness and divisiveness are consistent with prior social networks literature, especially with the concepts of ranked clusterability^11^, partitioning nodes via blockmodeling^3^, in-group attraction and out-group repulsion mechanisms^46^, and intra- and inter-group conflicts in small groups^47,48^, as well as sociological literature on faultline theory^49^. The theoretical underpinnings of our proposed methodology are discussed further in the Supplementary Information.

### Measuring balance at the macro-level

The line index of balance, denoted as *L*(*G*) and also referred to as the *frustration index*^7,9^ and *global balance*^21^, is defined as the minimum number of edges whose removal leads to balance. These edges can be thought of as sources of tension in this approach. While the historical roots of the frustration index go back to the 1950’s^5,50^, this approach only started to receive major attention in recent years^8,9,18,21,51^. This might be due to the computational complexity of obtaining this index exactly, which is an NP-hard problem^52^. While even approximating this measure has been difficult^21,51^, recent developments have enabled the exact and efficient computation of *L*(*G*) for graphs with up to $10^5$ edges^44,45^. To the best of our knowledge, the frustration index has not been previously applied to directed graphs. As a technical contribution, we have developed the first exact method for computing this index for directed signed graphs by building on recently proposed optimization models^18,45^.

Frustration of an edge depends on how the edge resides with respect to the partition $P=\{X,V{\setminus } X\}$ that is applied to *V*. Positive edges with endpoints in different subsets and negative edges with endpoints in same subset are frustrated edges under *P*. The frustration index offers a top-down evaluation mechanism for assessing partial balance by providing an optimal partition $P^*$. The optimal partition $P^*$ minimizes the number of frustrated edges and is therefore the best fitting partition of nodes into two mutually antagonistic and internally solidary groups. A simple normalization of *L*(*G*) using a line index upper bound (which equals a half of the edge count, *m*/2^44^) leads to the normalized line index $F(G)=1-2L(G)/m$^8^. The normalized line index provides values within the unit interval such that large values represent higher partial balance and therefore higher consistency of a network with balance theory at the macro-level. More details on this measure and the optimization model we use for this computation are provided in the Supplementary Information. We solve the optimization model that produces $P^*$ using *Gurobi* solver^53^ (version 9.0) in *Python*. For large networks, we follow the two-step method presented in^18^, which involves computing a lower bound for the frustration index before solving the optimization model.

## Results and discussion

### Measuring balance of static networks at three different levels

Our first two research questions ask to what degree our impression of the balance of a network depends on the chosen level of analysis (micro, meso, or macro). To answer these questions, we quantify balance for each level separately, and then base our interpretation of the balance of the network on the results across levels.

To measure balance of static signed directed networks at the micro-level, we use *T*(*G*) the proportion of balanced triads in a network among all transitive triads. Table 2 shows that triad-level balance values are high across all static networks with an average of 0.78 (Min = 0.52, Max = 0.90, SD = 0.12), except for the *College House B*, where only 52% of triads satisfy the semicycle balance property. Our results are consistent with the central tenet of balance theory, which states that networks strive towards stability in their triadic configurations, which then leads to high proportions of balanced triads and reduced tension^54^. Note that a micro-level measurement of balance based on triads may fall short in sparse networks where triads are infrequent and the clustering coefficient (the fraction of closed triples to all triples) is low^8^. Therefore, values of *T*(*G*) for *Reddit*, *Wikipedia*, *Bitcoin Alpha*, and *Bitcoin OTC* are more suitable to be interpreted as balance measurements of triads as opposed to that of the overall network sampled through the triads.

**Table 2:** Balance results for static networks.

Network	Density	Clustering coefficient	Triad level balance *T*(*G*)	Subgroup level balance		Network level balance *F*(*G*)	Triad census (transitive and balanced)
				Cohesiveness $C(P^*)$	Divisiveness $D(P^*)$		
Reddit	3.60E$-$4	6.30E$-$02	0.704	0.936	0.096	0.859	‘300’: 3.3%; ‘120D’: 10.3%; ‘120U’: 9.7%; ‘030T’: 47.1%
Wikipedia election	2.00E$-$03	5.30E$-$02	0.751	0.869	0.765	0.710	‘300’: 0.27%; ‘120D’: 6.1%; ‘120U’: 7.2%; ‘030T’: 61.5%
Bitcoin OTC	1.00E$-$03	4.50E$-$02	0.866	0.960	0.871	0.908	‘300’: 53.4%; ‘120D’: 7.7%; ‘120U’: 10.5%; ‘030T’: 15.0%
Bitcoin Alpha	1.70E$-$03	6.40E$-$02	0.845	0.960	0.781	0.909	‘300’: 61.4%; ‘120D’: 6.5%; ‘120U’: 10.8%; ‘030T’: 5.8%
Highland tribes	0.483	0.527	0.870	0.806	1.000	0.759	‘300’: 87%
College-House A	0.224	0.392	0.807	0.793	0.861	0.638	‘300’: 7.0%; ‘120D’: 19.3%; ‘120U’: 22.8%; ‘030T’: 31.6
College-House B	0.305	0.398	0.522	0.739	0.811	0.542	‘300’: 6.5%; ‘120D’: 2.2%; ‘120U’: 23.9%; ‘030T’: 19.6%
College-House C	0.213	0.271	0.896	0.909	0.973	0.877	‘300’: 3.4%; ‘120D’: 27.6%; ‘120U’: 10.3%; ‘030T’: 48.3%

**Figure 2:**
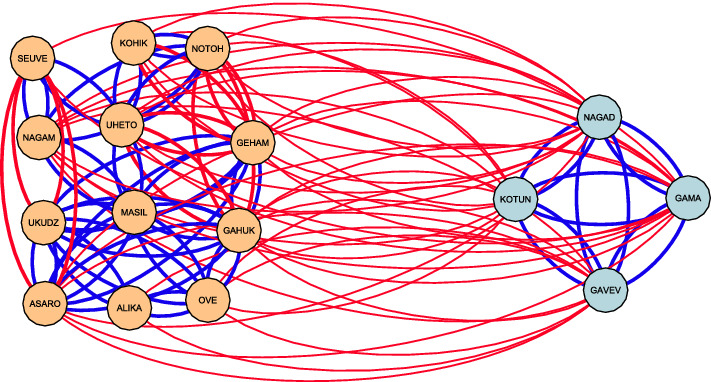
Visualization of optimal partition for Highland tribes network (low cohesiveness, high divisiveness). Direction of arcs is clockwise. Blue arcs are positive and red arcs are negative.

To evaluate balance of static signed directed networks at the meso-level, we compute our proposed measures of cohesiveness (intra-group solidarity) and divisiveness (inter-group antagonism). The numerical results for both metrics are provided in Table 2. We observe cohesiveness to be high with an average of 0.87 (Min = 0.74, Max = 0.96, SD = 0.08). Divisiveness is also high (except for the *Reddit* network), with an average of 0.77 (Min = 0.10, Max = 1.00, SD = 0.29). The meso-level values for most networks seem to indicate a positive association between the two measures. In other words, we observe high meso-level balance in networks in which nodes within the same subgroup are positively tied to their subgroup members, while at the same time, they are negatively tied to members of the other subgroup. This observation is consistent with literature on small-group conflicts, where strong in-group identity^55^ and weak out-group identity^48^ signify subgroup boundaries^47^. The *Reddit* network deviates from this general pattern as it shows high cohesiveness (0.936) and low divisiveness (0.096). While one might expect negative edges to dominate the ties between groups, the visualization of *Reddit* network (in the Supplementary Information) shows a prominence of positive edges (in blue) in general and between groups, which suggests the existence of one cohesive community rather than two divided subgroups for this particular network. Another deviation from the generally observed patterns is seen in the *Highland tribes* network, which has a lower value for cohesiveness compared to its high divisiveness. The visualization of this particular network (Fig. 2) shows the complete dominance of negative edges between subgroups, which explains the extreme divisiveness value ($D(P^*)$ = 1.00). However, negative edges are also present in one of the subgroups (13.5% of all edges in the left subgroup). In other words, while the two subgroups are clearly divided, there is also some division within one of the subgroups, which influences the overall cohesiveness of subgroups. A closer look at Fig. 2 shows that there are only two tribal alliances, ‘Masil’-‘Uheto’ and ‘Masil’-‘Nagam’, that keep the left subgroup together, while seven pairs of tribes in the left subgroup are mutually antagonistic. This lack of cohesion is accounted for by our measurement and consequently impacts the cohesiveness value.

To measure macro-level balance of static signed directed networks, we compute the normalized line index of balance. We find this index to be high with an average of 0.78 (Min = 0.54, Max = 0.91, SD = 0.14) for the static networks. The *College House B* network has the lowest normalized line index ($F(G) = 0.54$). Moreover, our results show that the values for *College House B* is the lowest at both the micro- and macro-level (but not at the meso-level). In other words, low proportion of balanced triads (52.2%) and a low normalized line index both suggest that tension is present in the network, and that we capture this effect consistently with different balance assessment methods. The *Bitcoin Alpha* network has the highest macro-level ($F(G) = 0.91$) and meso-level cohesiveness ($C(P^*) = 0.960$), and the fourth highest cohesiveness based on the the micro-level ($T(G) = 0.845$). This network shows a profile of balance across different levels, such that balance is reflected in high proportions of balanced triads, high cohesiveness within subgroups and medium-high divisiveness between subgroups, and relatively few frustrated edges in its optimal partitioning. Similar to the prior example, we conclude that this network is partially balanced, and our metrics consistently suggest this conclusion. Our method offers a profile of balance for distinguishing input networks with respect to balance at different levels instead of a single value.

### Insights from multilevel evaluation of balance in static networks

Our second research question asks about insights gained from applying our proposed multilevel evaluation framework to static signed directed networks. We observe similarities between micro- and macro-level balance when networks are dense and have high clustering coefficients. Consistent with observations from a previous study (see Fig. 2 in^18^), we find that aggregating micro-level balance results could represent macro-level balance when a network is densely connected and primarily consists of closed triads. On the other hand, for sparse networks, aggregating micro-level balance might not lead to similar results as conducting a macro-level evaluation since such networks cannot be reconstructed through the aggregation of their triads.

The *Reddit* network exemplifies this situation, with a low density of $3.6\mathrm{e}{-}04$ and a low clustering coefficient of $6.30\mathrm{e}{-}02$. While this network’s micro-level balance is 0.704, its numerous positive edges have led to a high cohesiveness of 0.936, and this effect then translates into a macro-level balance of 0.859. With lower intensity, a similar situation is observed for *Bitcoin Alpha* and *Bitcoin OTC* (both visualized in the Supplementary Information), which are also characterized by low density ($1.70\mathrm{e}{-}03$ and $1.00\mathrm{e}{-}03$, respectively) and low clustering coefficients ($6.4\mathrm{e}{-}02$ and $4.5\mathrm{e}{-}02$, respectively). The possibility for a large difference between balance in sparse networks when measured at the micro- versus macro-level has been also observed in prior work (see p. 23 in^8^). For the *Wikipedia* network (which also has low density and a low clustering coefficient), balance measures at the micro- and macro-level are similar. The consistency in balance values in the results above suggests that the sources of tension are well-represented in the triads of the network as well as in the overall macro-level structure. We conclude that while there are cases in which the two measurements match, balance at the micro- and macro-level is not generally the same property measured at different levels.

### Multilevel balance in temporal and multilayer networks

Our third research question asks about the generalizability of our methodology to evaluate balance of temporal and multilayer networks. To demonstrate the generalizability of our proposed framework, we apply it to two temporal networks (*Sampson’s monastery* and *Newcomb’s fraternity* and one multilayer network (*Collins philosophers*).

**Figure 3:**
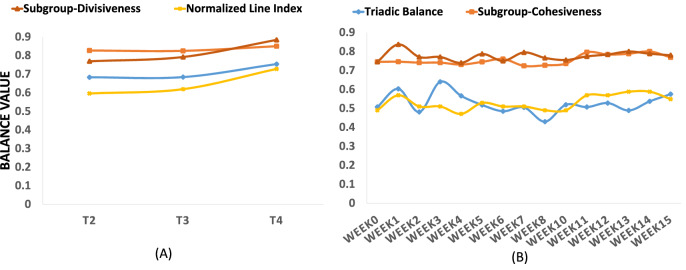
Multilevel balance values over time for Sampson’s affect (**A**) and Newcomb’s fraternity (**B**) networks.

**Figure 4:**
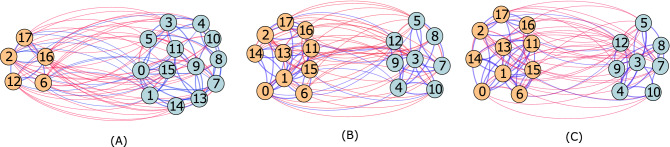
Visualization of optimal partitions for Sampson’s affect network over three snapshots, with (**A**) for T2, (**B**) for T3, and (**C**) for T4.

Measurements of balance for the two temporal networks, *Sampson’s monastery* and *Newcomb’s fraternity*, are shown in Fig. 3. For *Sampson’s monastery*, triadic balance (blue line) has an average value of 0.71. The average cohesiveness (orange line) is 0.83, while divisiveness (red line) has an average of 0.82. The normalized line index (yellow line) has an average value of 0.65. The overall trend is that measurements at all levels show increases in balance over time; supporting the basic premise of balance theory that networks tend to move towards balance^1,2,9,56^. Using optimal partitions, we also examine subgroup membership for the *Sampson’s monastery* network over time in order to analyze changes in how nodes form subgroups. We find that membership in the two subgroups changes from T2 to T3, and remains unchanged from T3 to T4 (shown in Fig. 4). Interpreting these results while considering the groups in Sampson’s study^31^ tells us that at T2, there is a smaller (blue) group of “outcasts”^31,57^, and a larger (yellow) group that consists of the dominant “young turks” and the “loyal oppositions” led by nodes 1 and 3, respectively. At T3, “outcasts” and “young turks” form one subgroup (yellow), while the “loyal oppositions” and “waverers” (those who did not identify with any faction) are in the other subgroup (blue). Interestingly, the optimal partitions remain unchanged in T4. According to Sampson^31^, the increase in polarization could be linked to the eventual disintegration of the monastery after T4, when some monks voluntarily left, while others (nodes 1, 2, 16, 17) were asked to leave.

Balance values for *Newcomb’s fraternity* network are shown in Fig. 3 (right). Micro-level values are, on average, 0.53 (Min = 0.43, Max = 0.64, SD = 0.05). Cohesiveness has an average of 0.75 (Min = 0.72, Max = 0.80, SD = 0.025), and divisiveness has an average of 0.77 (Min = 0.74, Max = 0.83, SD = 0.024). The normalized line index for macro-level balance has an average value of 0.53 (Min = 0.47, Max = 0.59, SD = 0.04). Interestingly, the low values for triadic balance and the normalized line index show that this network has an overall low level of partial balance when assessed at both the micro and macro level. Hence, this network’s ties are more in conflict with one another compared to most other networks considered in this paper. However, the process of inferring signed ties could impact balance values (as explained further in the Supplementary Information).

Similar to the patterns observed for *Sampson’s monastery* network, the *Newcomb’s fraternity* network has higher balance values at the meso-level and lower values at the micro- and macro-level. In this network, we do not see a clear temporal trend of increasing balance regardless of level of analysis (a time series decomposition did not show a trend). Instead, we see balance measurements oscillate around a narrow range of values over time, which is in contrast to observations by^56^ about another model of Newcomb’s fraternity data, which was later criticized in^58^. Our observation is consistent with previous studies suggesting that some networks have no clear tendency to move towards balance over time^59^. There is more variation over time in triadic balance and divisiveness compared to cohesiveness and normalized line index (as shown in Fig. 3B). Such differences suggest the presence of social mechanisms that only influence balance at certain levels, as captured by each different measurement of balance. In week 3, for example, there is a notable increase in micro-level balance, while balance at other levels remains the same. A closer look at the types of triads reveals a substantial increase in balanced ‘030T’ (which has one transitive semicycle) triads, and a substantial decrease in unbalanced ‘120D’ (which have two transitive semicycles) triads. One reason could be that, according to Newcomb^19^, friendships started to appear from week 3 onward. Friendship is a social precondition for reciprocity, transitivity, and balance at the dyadic and triadic levels, which may explain the observed increase in triadic balance in week 3.

Measurements of balance for the *Philosophers* multilayer network (visualized in the Supplementary Information) show changes in values depending on whether the two layers of the network (acquaintanceship and master-pupil) are analyzed separately or jointly. At the macro-level, for instance, the optimal partitions obtained separately for layers of the network are associated with four and six frustrated edges, while the optimal partition for the whole network (considering both layers) has 60 frustrated edges. This suggests that most sources of tension at the macro-level operate across layers. Specifically for this network, the two layers are interdependent in that three philosophers may be connected through a mix of both types of ties: master-pupil and acquaintanceship. When we analyze the two layers independently, we observe triadic balance values of 0.92 for acquaintance relations, and 0.95 for master-pupil relations, which largely indicate the absence of tension. When the two layers are combined into one flattened network, the triadic balance value decreases to 0.80. We observe a similar pattern of lower intensity of balance when analyzing the two types of relationships jointly instead of separately at the subgroup and network levels. Meso-level cohesiveness and divisiveness decrease from 0.99 and 0.98 to 0.97 and 0.93, respectively, and the normalized line index decreases from 0.98 to 0.94 when the two types of ties are considered simultaneously. These results indicate that the sources of tension involve groups of philosophers who were connected by a mixture of acquaintance and master-pupil relations and therefore manifest across layers of the network, such that they cannot be detected in either of the layers. Collins^32^ discussed that while master-pupil relationships may seem tension-free, in reality, how “ideas are created has always been a discussion among oppositions” (p. 1).

Methodologically, our observations when analyzing two temporal networks and one multilayer network show that the proposed multilevel framework not only allows for analyzing balance as a set of structural properties across three levels, but also for capturing dynamics of balance over time and across different layers of relations. Substantively, the observed temporal dynamics could show a network moving towards higher polarization (*Sampson’s network*) or a network without a monotone trend, oscillating over time within a short range of balance values (*Newcomb’s fraternity*). Our results for the multilayer network show that the co-existence of multiple types of signed ties may impact balance of a multilayer network in a more profound manner than the individual impact of each type of relation in its respective single-layer network.

### Methodological findings and implications

Several studies have compared triadic level balance with estimates for the line index of balance, and found that the line index (as a measure of unbalance) correlates with other measures of unbalance, such as the proportion of unbalanced triads^60,61^. Using normalized versions of triadic balance and the line index, high correlation is observed for networks with high density^8,18^, while measurements for sparse networks usually do not match^8^. However, the literature does not clarify whether such correlations are due to similarities in measurement or structural mechanisms in networks that yield similar balance values at different levels. Here, we briefly examine bivariate associations, if any, between triad-level, subgroup-level, and network-level balance and their associations with other network properties.

For the static networks, we find a positive and statistically significant correlation between micro-level balance and macro-level balance (Pearson’s $r=0.697, p<0.05$) (see the Supplementary Information for analytically obtained connections between the two measures). In addition, there is a positive correlation between meso-level cohesiveness and macro-level balance ($r=0.944, p<0.001$) (these two measures are both related to the optimal partition $P^*$). In the Newcomb’s fraternity temporal networks, we find positive and statistically significant correlations between macro-level balance and both meso-level balance measures (macro-level and cohesiveness: $r=0.849, p<0.001$; macro-level and divisiveness: $r=0.717, p<0.01$). These results demonstrate that the correlations among the balance measures could be complex, non-linear, and may depend on the network’s structural characteristics. In contrast to prior studies that suggest that micro-level balance induces macro-level balance^13,62^, our methodological framework deals with each balance measurement independently, and we do not claim any causality between these concepts. Rather, we speculate that there could be different social mechanisms that regulate balance at each level of analysis^27,63^. Further studies are needed to substantiate any potential causal links. The implication of our framework at this stage is to facilitate the evaluation of balance of directed signed networks at three levels of analysis with respect to the social processes involved at each level. Given the mixed observations and inconsistencies in empirical evaluations of balance, we consider this implication as a fundamental step towards a multilevel and multitheoretical framework^63^ for explaining the co-existence of distinct balance processes at the micro-, meso-, and macro-level.

## Conclusion

In this paper, we developed and applied a multilevel framework for the computational analysis of balance in signed directed networks. Our analysis of a variety of networks (including temporal and multilayer networks) representing various social settings (from college students and Wikipedia editors to philosophers and Bitcoin traders) shows that balance presents differently across multiple levels of the networks, leading to different profiles of social structures in triads, subgroups, and the whole network. In most cases, we observe relatively high values of balance across the three levels under consideration despite the differences in social setting and types of signed ties. Our study also serves as another confirmation of balance theory, with which the analyzed networks show a partial but considerable consistency. Our comprehensive numerical results suggest that values of balance at the micro-, meso-, and macro-level may match up to some extent. In the absence of other network dynamics, for which we have not tested, our findings suggest that the underlying mechanisms of avoiding tension and conflict may be reflected in micro-level patterns of balanced transitive semicycles. At a higher level, such patterns form a meso-level of internally cohesive and externally divisive subgroups. These effects eventually give rise to a macro-level polarization, where only a relatively few edges are positioned inconsistently with respect to the assertions of balance theory. We also find empirical evidence for the argument that balance at different levels is not just the same social phenomenon measured at different levels, but represents different properties (triadic balance, internal cohesion of subgroups, external division of subgroups, and overall polarization of the network), and should therefore be evaluated independently from one another. We have provided empirical evidence which suggests that these distinct structural properties of signed social networks are inextricably intertwined by a set of fundamental rules of partial balance. These fundamental rules could be expressed at each of the three levels of the networks: (1) In networks partially balanced at the micro-level, balanced triads outnumber unbalanced triads. (2) Networks partially balanced at the meso-level have an optimal partition with two internally cohesive and externally divisive subgroups. (3) Networks partially balanced at the macro-level are a relatively few number of edges away from balance.

Our study is an intermediate step that advances our knowledge about the structure of signed social networks. The generalizability of our findings is subject to certain limitations. First, we analyze networks with up to 120,000 edges (upper bound due to the demanding complexity of our exact computational methods). We hope to extend this multilevel framework to an even wider range of social networks before confirming what this study has partially substantiated for the first time. Second, we aim to include more temporal and multilayer networks, combining the two in future studies in order to generalize our observations with respect to how balance at different levels may change over time and across types of relationships.

## Materials and methods

We analyze 11 networks that have ties with signs and directionality. These data are publicly available for research purposes^33^. Here, we briefly outline the context and collection of primary data, and how they were processed into network data. Technical details on network data pre-processing are provided in the Supplementary Information.

### Static networks

Most of the static networks in our data were previously used for network-related research. Specifically, the *Reddit* dataset^34^ represents content with negative sentiment that was exchanged between posts from two different communities of users (subreddits). *Wikipedia election* network^35^ was collected to study the relationship between sentiment and person-to-person evaluation of leadership and credibility. Hence, the data represent approval or disapproval of voters for deciding whether to promote a user to be a Wikipedia administrator. *Bitcoin OTC* and *Bitcoin Alpha* were both built from user ratings of trust towards other users on the Bitcoin online trading platforms. The authors^36^ used the data to build a prediction model for edge weights using measures of trust between individuals. In contrast to these datasets, which were collected from online sources, *Highland tribes* and *College Houses A, B, C* data were collected through fieldwork and surveys, respectively. *Highland tribes*^37^ was created through fieldwork that included observations and conversations with political leaders and tribal members of the Central Highlands in New Guinea. The signed network represented political alliances and oppositions among 16 tribes. Network data for College Houses A, B, C^17^ come from a small group survey on evaluations of other group members (sorority sisters) based on a range of behavioral characteristics.

### Temporal and multilayer networks

Two temporal networks, *Sampson’s affect* and *Newcomb’s fraternity*, were both collected for qualitative social network analysis. *Sampson’s affect*^31^ was collected via 12 months of fieldwork at an American monastery, where interactions between 18 monks were recorded. The author also created a questionnaire asking each monk to list “3 brothers who you like the most” in order to examine the friendship network among different subgroups of monks (young turks, outcasts, loyal oppositions, and waverers). *Newcomb’s fraternity* data was created from a 15-week survey of 17 fraternity brothers at the University of Michigan in 1956. The temporal nature of the dataset allowed for in-depth examination of the formation of acquaintanceship and social groups. For the *Philosophers* network data, master-pupil and acquaintanceship ties between philosophers from 800 B.C.E to 1935 C.E were gathered from Randall Collins’ seminal book^32^ based on close readings of historical texts. Among many findings, through an examination of the ties between different groups of philosophers, the author found that successful philosophers had the most ties to other philosophers, regardless of the signs of ties.

## Supplementary information

Supplementary Information 1.

Supplementary Information 2.

Supplementary Information 3.

Supplementary Information 4.

Supplementary Information 5.

Supplementary Information 6.

## Data Availability

All network data and code used in this study are made publicly available. Links and descriptions for data and code are provided in the Supplementary Information.
