# Supplementary material for: Multilevel structural evaluation of signed directed social networks based on balance theory
Source: Sci Rep. 2020 Sep 17;10:15228. doi: 10.1038/s41598-020-71838-6 (PMC7498592; doi:10.1038/s41598-020-71838-6)
Supplement: Supplementary file 1 — Supplementary Information 1. [file 41598_2020_71838_MOESM1_ESM.pdf]

Multilevel Structural Evaluation of Signed Directed Social Networks based on Balance Theory

Samin Aref[1,+,*], Ly Dinh[2,+,*], Rezvaneh Rezapour[2,+,*], and Jana Diesner[2]

[1]Laboratory of Digital and Computational Demography, Max Planck Institute for Demographic Research, 18057, Rostock, Germany
[2]School of Information Sciences, University of Illinois at Urbana-Champaign, Champaign, USA
[*]Corresponding authors: aref@demogr.mpg.de, {dinh4,rezapou2}@illinois.edu
[+]These authors contributed equally to this work. Authors are listed in alphabetical order.

This document describes all supplementary information for the article "Multilevel Structural Evaluation of Signed Directed Social Networks based on Balance Theory".

This PDF file includes:

Supplementary Text
Detailed Materials and Methods
Figs. S1 to S6
Tables S1 to S2
Captions for Movies S1 to S3
Captions for Databases S1 to S3
References 64-65

Other Supplementary Materials for this manuscript include the following:

Movies S1 to S3
Databases S1 to S3

Supplementary Text

Data availability

All network data and numerical results related to this study are publicly available through links provided in this document. The code for the computational analysis including the optimization models used is this study is made publicly available on the Github repository https://github.com/saref/multilevel-balance upon publication of the paper.

Additional information about the networks

In this subsection, we provide detailed descriptions of all the data sources used in the study to facilitate contextualization of the actual real-world system or group that is modeled as a signed directed network.

The Reddit network represents directed signed ties of communication between two users (belonging to different subreddits), inferred through sentiments of multiple posts and comments they have exchanged. Each connection (edge) consists of three different attributes: the time, the sentiment of the source post, and the text vector of the source post. The network spans approximately 2.5 years (Jan 2014 to April 2017) and includes all the public messages shared on Reddit during that time period[34].

The Wikipedia election data consists of all votes (supporting, neutral, or opposing) in elections for promotion of a user to admin role (when a user requests promotion to adminship). The network includes all votes that were cast from 2003 through May 2013[35].

The Bitcoin OTC and the Bitcoin Alpha datasets both represent trust between Bitcoin traders on two platforms, called Bitcoin OTC and Bitcoin Alpha. The transactions on these platforms are anonymous. However, to minimize fraudulent behavior and to maintain reputation, members of OTC and Alpha rate others in a scale of -10 (total distrust) to +10 (total trust) in steps of 1[36].

The Highland tribes dataset represents an alliance structure among tribal groups using the Gahuku-Gama system of the Eastern Central Highlands of New Guinea. The dataset consists of two parts: (1) GAMAPOS representing alliance ("rova") relations, and (2) GAMANEG for antagonistic ("hina") relations[37].

The college A, B, and C datasets are constructed with respect to the relationships between three different groups of college girls at an Eastern college in the United States. Each group consists of approximately 20 members who lived together for at least four months. The girls in each house were asked to provide evaluations of other girls in the same house based on a range of behavioral characteristics. These data are then converted into matrices of choice and rejection for each house[17].

The Sampson monastery network represents positive and negative relationships between 18 monks (novices) in a monastery in the United States. To construct the social network, each monk was asked to rank his top three choices with respect to four positive social relationships. In addition, they were asked to create the same list for negative relationships. The data is collected at five different times, but the group we analyze were present only in three data collection times[31].

The Newcomb's fraternity dataset represents the relationships between 17 boys living in a pseudo-dormitory within a U.S. college. The members of the dormitory were asked to rank their relationship with other people over the period of 15 weeks. The top and bottom preference rankings are assumed to represent positive and negative relationships respectively[19].

The Philosophers network is constructed based on Randal Collin's collection of historical data in his seminal work "The Sociology of Philosophies"[32], in which he studies various types of philosophical thoughts in ancient Greece, China, Japan, India, the medieval Islamic and Jewish world, medieval Christendom, and modern Europe. The book represents the divisions and conflicts among philosophers based on acquaintanceship relationships and relationships between masters and pupils.

From the micro-level to the macro-level of balance

The graph-theoretical background of balance theory shows a simple, but often disregarded limitation for evaluating balance merely based on triads of a network; balance of a network's triads is not a sufficient condition for balance of the network. Some researchers have circumvented this issue in undirected signed graphs by arguing that cycles longer than 3 are not particularly important[42] and continued using triads on the basis that their balance is a necessary condition for balance of a network. In digraphs, however, both of these premises fall apart, which accentuates the problem. In an unbalanced digraph, all semicycles of length 3 and above could be balanced if an unbalanced semicycle of length 2 exists. Balance in transitive semicycles of length 3 is not the only condition for balance of a signed digraph. In our proposed methodology, we have also used the line index of balance which provides a complementary perspective on balance compared to what is achievable by using only semicycles and triads. Two key connections between our micro-level and macro-level measures are that every unbalanced triad has at least one frustrated edge[45], and that the frustration index equals the minimum number of unbalanced fundamental cycles induced over all spanning trees of the graph[51].

Theoretical underpinnings of our approach

Our multilevel framework of structural balance evaluation is motivated by an extensive pool of literature from network science[3,5,8,9] and organizational sciences[47–49]. The literature recognizes that networks inherently consist of multiple levels[63], in which different social forces and structural processes that could influence balance dynamics co-occur. We begin our multilevel framework with a triadic analysis, primarily because triads are often assumed to be building blocks of networks[11] where structural dynamics involving signs and directionality take place. Indeed, earliest formulation of structural balance was made by Heider[1] at the triadic level (2 persons and a common object). Heider developed a typology of triads which included four sign configurations of balanced triads (with 0 or 2 negative edges), and four sign configurations of unbalanced triads (with 1 or 3 negative edges). Latter efforts expanded these triadic configurations to consider directionality of edges[11,14–16]. This subset of literature leveraged semicycles and posited that a signed directed triad is "balanced if and only if all semicycles have positive signs"[11]. Johnsen[38], followed by Holland and Leinhardt[41], then developed the triad census that contained all possible configurations of balanced and unbalanced triads, considering both signs and directions of edges in semicycles.

At the same time, several network science scholars extended structural balance measures to include groupings beyond three people (a triad). Cartwright and Harary[2] derived the *structure theorem* that posited a signed network to be balanced if it is possible to subdivide all nodes into two sets in ways that all positive edges occur within the same set, and all negative edges occur between sets. Doreian and Mrvar[3] proposed a similar framework to partition nodes into distinct *plus-sets*. Davis and Leinhardt[4] then proposed a ranked clusterability model that grouped dyads together in the same cluster if they both have positive and reciprocated edges, and separated dyads into two clusters if they both have negative and reciprocated edges. The structural notion of positive ties within subgroup and negative ties between subgroups was also widely examined in studies of teams[47,48] and organizational communication[49]. A study of team trust by Ashleigh and Stanton[55], for example, found that most teams have stronger trust scores at the intra-team level than the inter-team level, primarily because members have already established a strong in-group identity and thus view working with the out-group members as a competitive task. Lau and Murighan[49] observed the emergence of "faultlines" in work-groups based on demographic attributes such as gender, age, and occupational roles. These faultlines produced homogeneous subgroups in which individuals have strong internal cohesion but weak external connections with other subgroups.

While triad-level and subgroup-level analyses capture the fine-grained dynamics that may influence balance between groups of nodes, there is a need to examine balance at the network-level to capture structural forces that influence balance beyond the groups and at a holistic level. Harary[5] proposed line index of balance to determine the smallest number of edges whose change of sign would result in a balanced network. The normalized version of this measure suggested in[8] expands the concept of a network's balance from being a binary state (either balanced or unbalanced) to a continuous scale for the level of balance according to which a network can be partially balanced[8,9].

Notes on common properties of the signed social networks

As a result of conducting macro-level balance assessment by computing its frustration index, signed networks will fall into one of three possible categories of partial balance: (1) close to balance, (2) random pattern of signed ties, and (3) far away from balance. In the first group, the frustration index is relatively small compared to its theoretical upper bound $m/2$[44], which leads to $F(G)$ being in the range of $(0.7, 1]$. Therefore, the network is just a few edges away from balance. In the second group, the frustration index is neither small nor large when compared to $m/2$, such that $F(G)$ takes values around 0.5. Random signed graphs were observed to show such behavior[8], where the frustrated index is roughly half of its maximum possible value $m/2$. In the third group, $F(G)$ takes values from $[0, 0.3)$, because signed ties are arranged such that the overall network is far away from balance, and a large number of edges (compared to $m/2$) would need to be removed to achieve balance. An extreme example is a complete signed digraph in which every pair of nodes is connected by one positive and one negative tie. In such a network, at least half of the edges would need to be removed to achieve a balance network ($L(G) = m/2, F(G) = 0$).

Given that a majority of the networks we analyzed represent high values of balance in different levels, one may wonder if the networks were selected only if they show consistency with balance theory. This is not the case, as we have included all publicly available signed directed network data that we were able to find from social domains. Therefore, observing high partial balance in all these networks, despite their difference in context, is yet another empirical confirmation for balance theory[9,21] and specifically how signed social networks represent a high level of partial (as opposed to total) balance[8].

Another possible concern could be that our methods of measurement are not calibrated and therefore only provide values in the higher half of the range $[0, 1]$, regardless of what the network is. This is not the case, as evidenced by the axiomatic comparison of balance measures in[8] and our testing of these measures on a different static network. *Chess network*[64] is a signed digraph produced from real data that represent chess players as nodes and the game outcomes as signed directed edges from the white player to the black player, with a positive (negative) sign for white victory (defeat). While this network comes from a real data-generation process, there is no social meaning to the ties, and we would not expect the network to represent high partial balance (consistency with balance theory). Confirming our intuition, triad level balance is measured at 0.47 and the network

level balance is 0.4 for the Chess network (both values showing a random pattern in signed ties), which shows that the two measures could distinguish between networks by providing high or low values as required.

Difference and relevance of micro- and macro-level balance

Despite support for the argument that balance in micro and macro levels are not generally the same property measured at different levels, there are special cases in which the micro and macro balance cannot be separated at all, as if they are indeed the same property. A fully connected signed network (which has a density and a clustering coefficient of 1) is balanced if and only if its triads are balanced[43]. Therefore, one could argue that in complete networks, not only the aggregation of balance in micro-level represents the balance of the overall structure, but balance at either micro- or macro-level is not mathematically possible without balance at the other level.

Uniqueness and multiplicity of optimal partitions

An essential part of our analysis of the networks has been to determine the optimal partitions, which is the first step for the meso-level and macro-level evaluation of balance. Previous studies on frustration index[9,18,44,45] do not discuss the multiplicity of optimal partitions. We configure the solving strategy of the mathematical model in Eq. (1) to not just find one optimal solution, but to systematically search through the space of solutions (feasible space) and find all optimal solutions. This involves finding all partitions whose frustration count equals the frustration index.

This further analysis requires intensive computations and can only be done for a relatively small network. Solving our proposed optimization model with this new search strategy allows us to confirm that the optimal solution is unique in *highland tribes*, all time-frames of *Sampson monastery* and *college preferences in houses B and C*. In contrast, we find that for *college preference house A*, the optimal solution is not unique and has a multiplicity of 3 (there are three partitions whose frustration count equals the frustration index). In Figure S1, the three optimal partitions of *college preference house A* are visualized.

While the number of frustrated edges in all three optimal partitions are equal, there could be differences in the composition of positive and negative edges among the frustrated edges in optimal partitions, which in turn could impact our measurements of cohesiveness and divisiveness.

Table S1. Multiple optimal partitions for college preferences in House A and values of cohesiveness and divisiveness

Optimal partition	X^*	Cohesiveness $C(P_i^*)$	Divisiveness $D(P_i^*)$
P_1^*	{4,8,10,15,16,18, 3,9,11}	0.804	0.842
P_2^*	{4,8,10,15,16,18, 9,11,19}	0.793	0.861
P_3^*	{4,8,10,15,16,18, 19}	0.793	0.861

In *college preference house A* network, the subset assignment of all nodes is consistent across multiple partitions except for four nodes: 3,9,11,19. As can be seen in Table S1 and Figure S1, these four nodes belong to different subsets across the three optimal partitions, while nodes 9 and 11 are always together and nodes 3 and 19 are always apart.

The values of cohesiveness and divisiveness are the same for P_2^*, P_3^* because their composition of positive and negative frustrated edges are the same, but for P_1^* there is a small difference in the two measurements. The three optimal partitions of *college preference house A*, lead to a small standard deviation of 0.006 between the cohesiveness values and a standard deviation of 0.011 between the divisiveness values.

This observation shows that there are networks in which the position of some nodes could be precarious across the two optimal subsets. For nodes 9 and 11 (connected by two positive arcs), membership in either of the two subsets is possible as long as they stick together. For nodes 3 and 19 (connected by a negative arc), optimal group assignments place them in different groups, but they all keep the two nodes separated. No possible benefit (in terms of reduction in frustration count) exists in the network that would offset the cost of separating 9 and 11 or the cost of placing 3 and 19 together.

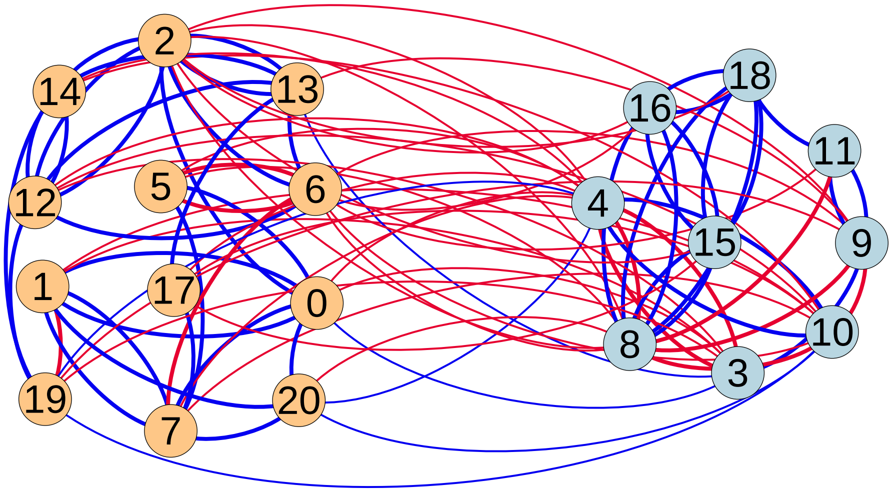

Optimal Partition P_1^*

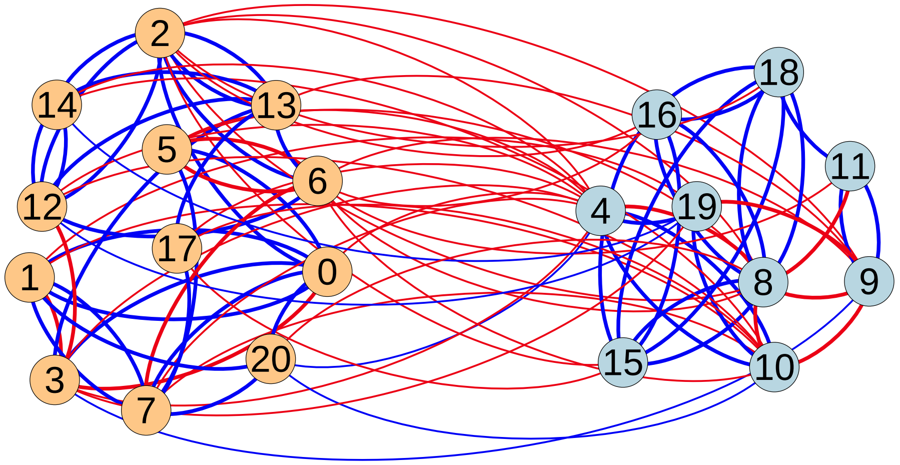

Optimal Partition P_2^*

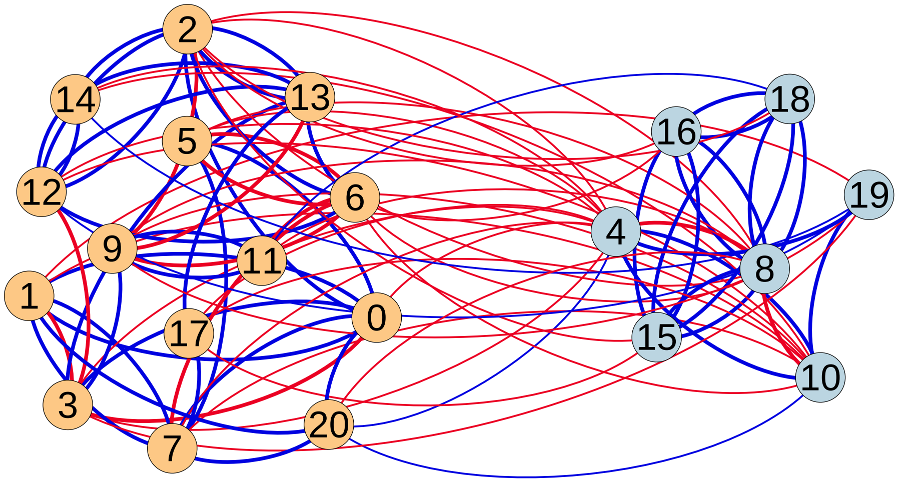

Optimal Partition P_3^*

Figure S1. Three distinct optimal partitions achievable for college preferences in House A. Direction of arcs are clockwise. Blue arcs are positive and red arcs are negative.

Detailed Materials and Methods

Processing of secondary data into network data

In this subsection, we provide detailed descriptions of data preprocessing for balance analysis of the relevant static networks, temporal networks, and multilayer network. All datasets are publicly available, and most are available through major network data repositories such as UCINET IV, Stanford Network Analysis Project (SNAP), and Index of Complex Networks (ICON). The exceptions were *Sampson's affect data*[31], *College Houses A,B,C*[17], and *Philosophers network* which were not in digitized form originally. We digitized Sampson's affect data from Tables in [3, p. 160-161] considering all the edges. We reconstructed the *College Houses* networks from sociomatrix Tables in [17, p. 18-20] such that all choices and rejections that had a weight of $-4, -3, +3, +4$ were considered as directed signed edges. Collins *Philosophers* network was digitized by Wouter de Nooy and provided to the authors.

Static networks: For four networks (*Reddit*, *Wikipedia election*, *Bitcoin OTC*, *Bitcoin Alpha*) we kept the original datasets without much preprocessing, given that the edge-lists were directed, and contained signed attributes (-1 or $+1$ in *Reddit* and *Wikipedia*, and -10 to $+10$, excluding 0, in *Bitcoin OTC* and *Bitcoin Alpha*). The strength of the edges were not considered and only the signs were imported for creating the networks. For *College Houses A, B, C*, the sign attribute contained values from -4 to $+4$. We only included edges that contain scores of -3 and -4, which we relabel to -1. and scores of $+3$ and $+4$, which we relabeled to $+1$. Lastly, for the *Highland tribes* network, we combined two edge-lists, *GAMAPOS* ($+1$) and *GAMANEG* (-1), which contains positive and negative relations among tribes, respectively. We then symmetrized the edge-list to convert the network from an undirected to a directed representation.

Temporal networks: For *Sampson's affect*, we used the the data we had digitized from Tables in [3, p. 160-161], which had a weight range from -1 to -3 and from $+1$ to $+3$, but we discard the strength of the edges and loaded the edges only based on their signs.

For *Newcomb's Fraternity*, the original dataset contains 15 matrices on the preference ranking from 17 men. Each person provides a score from 1-16 for every other person in the fraternity house, in decreasing order of their preference for friendship with that person. To create a directed signed network, we filtered the data to include only the reported top 3 ranks and bottom 3 ranks (which we considered as positive and negative edges respectively). We did not consider different strengths for the edges. For *Newcomb's fraternity* data, we created signed graphs with edges based on the three least (most) preferred nominations of each person as their enemies (friends). For negative ties, in particular, this choice may be contestable, though it is consistent with the balance analysis of the same data in[56]. Our preliminary post-hoc analysis, which uses different choices for inferring signed ties (e.g., top and bottom 1 ranks, top and bottom 2 ranks), reveals notably different balance values. Therefore, further investigations on modeling signed network data are required to better understand and interpret signed networks inferred from preference ranking data.

Multilayer network: For *Philosophers network*, we considered edges with weights of -2 and $+2$, representing negative and positive edges between philosophers, respectively. We discarded edges with weight of 1, as they represent "probable ties" according to[32]. We then symmetrized all edges of the network in the two layers (master-pupil and acquaintanceship), and combined the two layers to create a flattened representation of the multilayer network.

After all network edge-lists were constructed, we pre-processed the data using *pandas* in Python. This step included calculation of triad-level balance and descriptive metrics such as global clustering coefficient, density, and triad census.

Obtaining optimal partition of directed signed graphs

Following recently developed computational methods[45], we formalizes the process for obtaining a globally optimal partition for a signed digraph as a binary linear programming model in Eq. (1), which computes the frustration index of input digraph G in its optimal objective function.

$$
\begin{aligned}
\min_{x_i, f_{ij}} Z = \sum_{(i,j)\in E} f_{ij} & \\
\text{s.t.} \quad f_{ij} \geq x_i - x_j \quad & \forall (i,j) \in E^+ \\
f_{ij} \geq x_j - x_i \quad & \forall (i,j) \in E^+ \\
f_{ij} \geq x_i + x_j - 1 \quad & \forall (i,j) \in E^- \\
f_{ij} \geq 1 - x_i - x_j \quad & \forall (i,j) \in E^- \\
x_i \in \{0,1\} \quad & \forall i \in V \\
f_{ij} \in \{0,1\} \quad & \forall (i,j) \in E
\end{aligned}
\tag{1}
$$

The optimization model formulated in Eq. (1) has one binary variable for each vertex and one binary variable for each edge of the digraph G. Binary variable x_i is associated with node i and represents the subset that node i belongs to (whether $i \in X$ or

not as a binary value). Binary variable f_{ij} is associated with directed edge (i,j) and represents its frustration state (whether directed edge (i,j) is frustrated or not as a binary value).

The objective function is the minimum of the total count of frustrated edges over the set of edges E. The first two constraints ensure that positive edges $(i,j) \in E^+$ are counted as frustrated if their endpoints i and j belong to different subsets. Similarly, the next two constraints handle the frustration of negative edges $(i,j) \in E^-$ when their endpoints i and j belong to the same subset. We solve the optimization model in Eq. (1) using *Gurobi* solver[53] (version 9.0) in *Python* and for large networks we follow the two-step method discussed in[18].

Computational analysis and solving the optimization model

The codes for both the computational analysis and optimization models are made available on a Github repository at `https://github.com/saref/mutlilevel-balance` once this paper is published.

The code for multilevel evaluation of balance, including optimization models, are distributed under an Attribution-NonCommercial-ShareAlike 4.0 International (CC BY-NC-SA 4.0) license. This means that one can use these algorithms for non-commercial purposes provided that users include proper attribution by citing[44,45] and the current article. Copies or adaptations of the algorithms should be released under the similar license.

For the computational analysis at the micro level, our algorithm lists graph triads and checks their transitivity. If all semicycles of a triad are transitive, then their balance is evaluated and provided as the output.

The proposed optimization model can be solved by any mathematical programming solver which supports 0/1 linear programming (binary linear) models. In the Github repository `https://github.com/saref/multilevel-balance`, we explain using the Gurobi solver (version 9.0) for solving the proposed model.

Our proposed optimization algorithm was developed in Python 3.7 based on mathematical programming models developed in[18,44,45], but it computes the frustration index of a directed signed graph.

The following steps outline the process for academics to install the required software (*Gurobi* solver[53]) on their computer in order to run the optimization algorithms:

1. Download and install Anaconda (Python 3.7 version), which allows you to run a Jupyter code. It can be downloaded from `https://www.anaconda.com/distribution/`. Note that you must select your operating system first, then download the corresponding installer.

2. Register for an account on `gurobi.com/registration-general-reg/` to obtain a free academic license for using Gurobi. Note that Gurobi is a commercial software, but can be registered with a free academic license if the user is affiliated with a recognized degree-granting academic institution. This involves creating an account on the Gurobi website in step 5.

3. Download and install the Gurobi Optimizer (versions 9.0 and above are recommended), which can be downloaded from `https://www.gurobi.com/downloads/gurobi-optimizer-eula/`, after reading and agreeing to Gurobi's End User License Agreement.

4. Install Gurobi into Anaconda. Do this by first adding the Gurobi channel to your Anaconda channels, and then installing the Gurobi package from this channel.

   From a terminal window, issue the following command to add the Gurobi channel to your default search list

   ```
   conda config --add channels
   http://conda.anaconda.org/gurobi
   ```

   Now issue the following command to install the Gurobi package

   ```
   conda install gurobi
   ```

5. Request an academic license from `gurobi.com/downloads/end-user-license-agreement-academic/` and install the license on your computer, following the instructions given on Gurobi license page.

   Completing these steps is explained in the following links (for version 9.0):

   for Windows `https://www.gurobi.com/documentation/9.0/quickstart_windows/installing_the_anaconda_py.html`,

   for Linux `gurobi.com/documentation/9.0/quickstart_linux/installing_the_anaconda_py.html`, and

for Mac `gurobi.com/documentation/9.0/quickstart_mac/installing_the_anaconda_py.html`.

After following the instructions above, open Jupyter Notebook, which will take you to an environment (a new tab on your browser will pop up) where you can open the main code (which is a file with .ipynb extension).

Additional numerical results for the networks

Table S2 provides all the numerical results and additional measurements produced for the network.

Table S2. Additional numerical results for the networks

Dataset	n	m	m^+	m^-	B.T.	U.T.	T(G)	C.C.	Density	$L(G)$	$F(G)$	$C(P^*)$	$D(P^*)$
Tribes	16	116	58	58	59	9	0.87	0.527	0.483	14	0.759	0.806	1
Reddit	18313	120792	111891	8901	237148	99546	0.704	6.30E-02	3.60E-04	8511	0.859	0.936	0.096
Wikipedia	7118	103675	81318	22357	426215	141380	0.751	5.30E-02	2.00E-03	15045	0.71	0.869	0.765
Bitcoin Alpha	3783	24186	22650	1536	11649	2141	0.845	6.40E-02	1.70E-03	1098	0.909	0.96	0.781
Bitcoin OTC	5881	35592	32029	3563	19447	2969	0.866	4.50E-02	1.00E-03	1644	0.908	0.96	0.871
House A	21	94	51	43	46	11	0.807	0.392	0.224	17	0.638	0.793	0.861
House B	17	83	41	42	24	22	0.522	0.398	0.305	19	0.542	0.739	0.811
House C	20	81	41	40	26	3	0.896	0.271	0.213	5	0.877	0.909	0.973
Sampson T2	18	104	55	49	41	19	0.683	0.349	0.34	21	0.596	0.827	0.769
Sampson T3	18	105	57	48	52	24	0.684	0.445	0.343	20	0.619	0.825	0.792
Sampson T4	18	103	56	47	49	16	0.754	0.412	0.337	14	0.728	0.85	0.884
Fraternity 00	17	102	51	51	38	37	0.507	0.376	0.375	26	0.49	0.745	0.745
Fraternity 01	17	102	51	51	35	23	0.603	0.406	0.375	22	0.569	0.746	0.837
Fraternity 02	17	102	51	51	26	28	0.481	0.406	0.375	25	0.51	0.741	0.771
Fraternity 03	17	102	51	51	32	18	0.64	0.376	0.375	25	0.51	0.741	0.771
Fraternity 04	17	102	51	51	47	36	0.566	0.435	0.375	27	0.471	0.731	0.74
Fraternity 05	17	102	51	51	44	41	0.518	0.492	0.375	24	0.529	0.745	0.787
Fraternity 06	17	102	51	51	50	53	0.485	0.484	0.375	25	0.51	0.76	0.75
Fraternity 07	17	102	51	51	50	49	0.505	0.525	0.375	25	0.51	0.724	0.795
Fraternity 08	17	102	51	51	40	53	0.43	0.543	0.375	26	0.49	0.727	0.766
Fraternity 10	17	102	51	51	40	37	0.519	0.461	0.375	26	0.49	0.736	0.755
Fraternity 11	17	102	51	51	37	36	0.507	0.467	0.375	22	0.569	0.796	0.774
Fraternity 12	17	102	51	51	38	34	0.528	0.486	0.375	22	0.569	0.784	0.784
Fraternity 13	17	102	51	51	44	46	0.489	0.516	0.375	21	0.588	0.788	0.8
Fraternity 14	17	102	51	51	44	38	0.537	0.475	0.375	21	0.588	0.8	0.788
Fraternity 15	17	102	51	51	50	37	0.575	0.498	0.375	23	0.549	0.769	0.78
Master-pupil layer	712	1354	1264	90	18	1	0.947	0.038	0.003	4	0.994	0.998	0.988
Acquaintance layer	346	660	474	186	72	2	0.917	0.091	0.006	6	0.982	0.996	0.979
Philosophers flat	855	2010	1736	274	78	19	0.804	0.089	0.003	60	0.94	0.976	0.931

B.T.: Balanced triads, U.T.: Unbalanced triads, C.C.: Clustering coefficient

Visualization of optimal partitions in large networks (Figures S2 to S6)

Fig. S2 shows the *Philosophers* network as a multilayer network, which is visualized using *MuxViz*[65]. Figs. S3–S6 show the optimal partitions in Bitcoin-Alpha, Bitcoin-OTC, Wikipedia, and Reddit respectively. The level of balance at the macro level is high in all these networks. While high macro-level balance is associated with high cohesiveness and high divisiveness (as in Figures S3–S5), this does not necessarily have to be the case (as shown in Figure S6. It can be visually observed that there is very low divisiveness between the subgroups of Reddit in Figure S6.

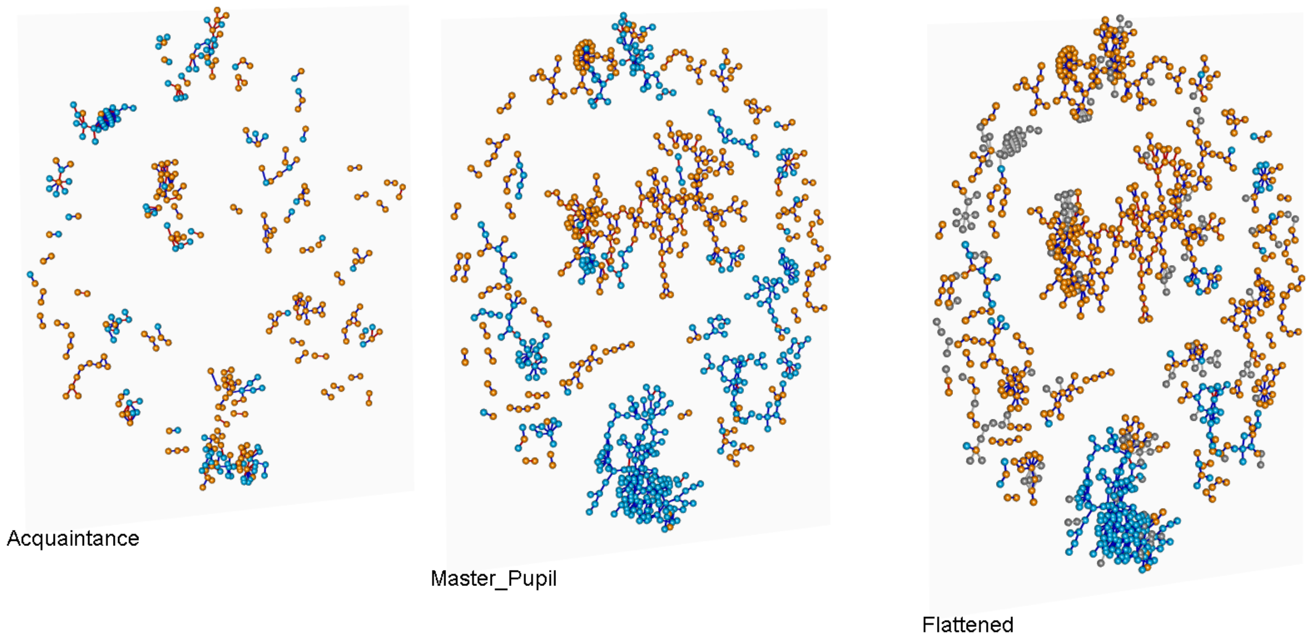

Figure S2. Two layers of the philosophers network and its flattened view

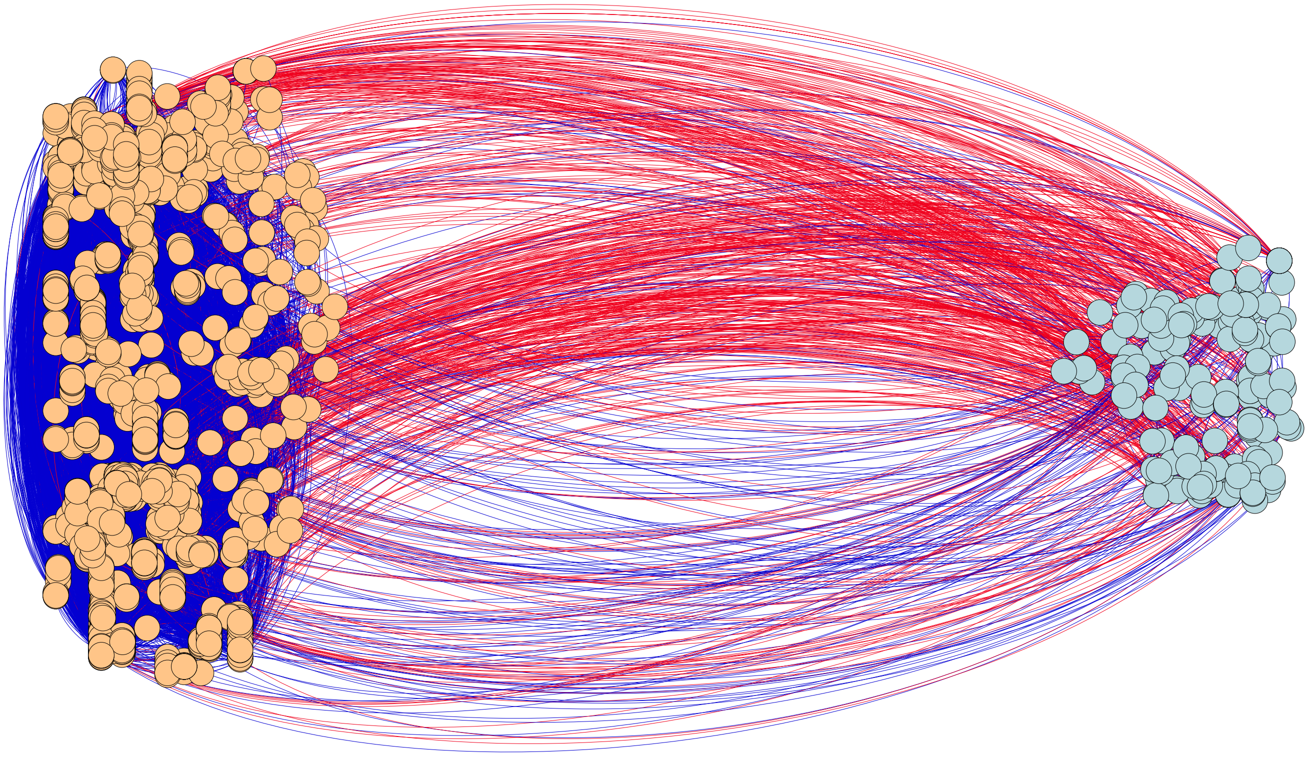

Figure S3. Visualization of Bitcoin-Alpha network and its optimal partition (high cohesiveness, high divisiveness)

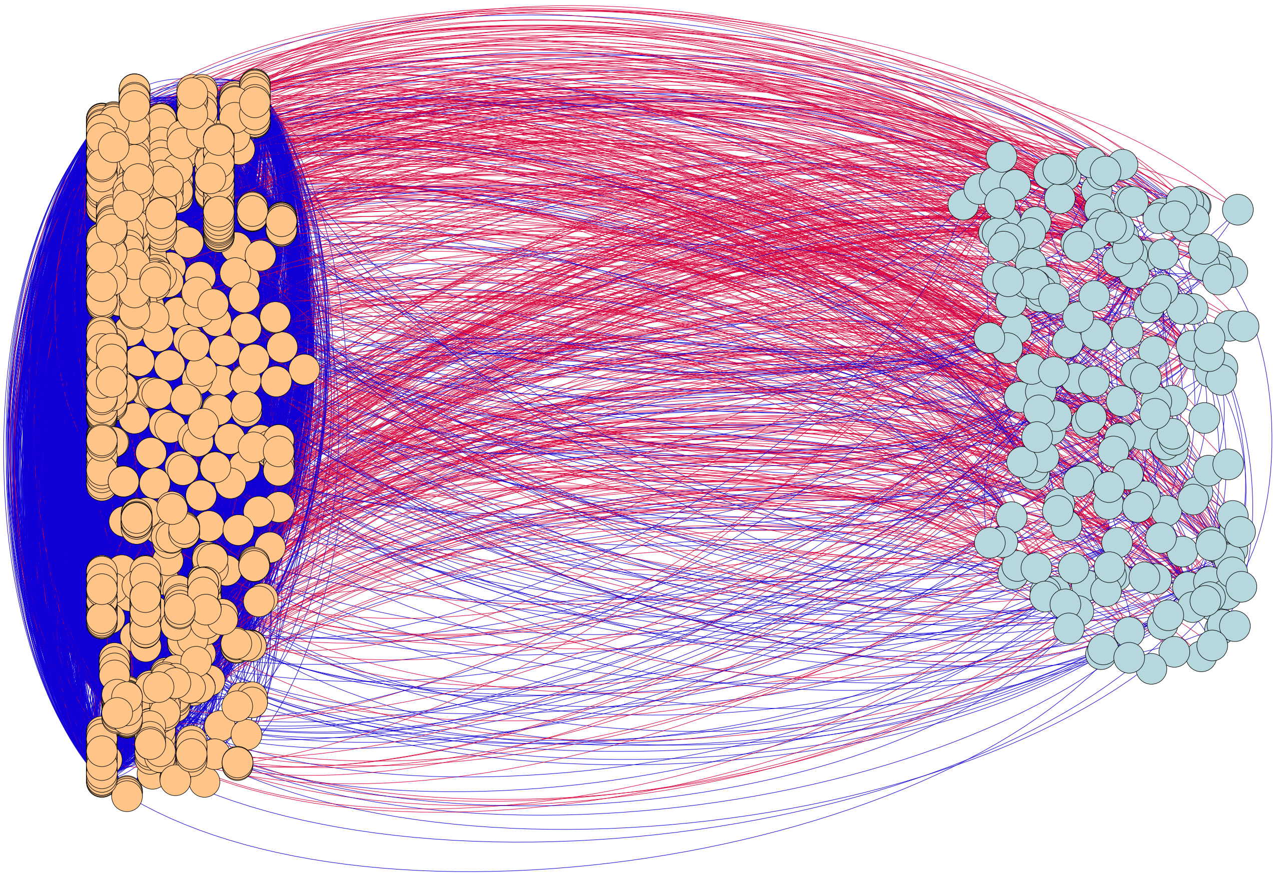

Figure S4. Visualization of Bitcoin-OTC network and its optimal partition (high cohesiveness, high divisiveness)

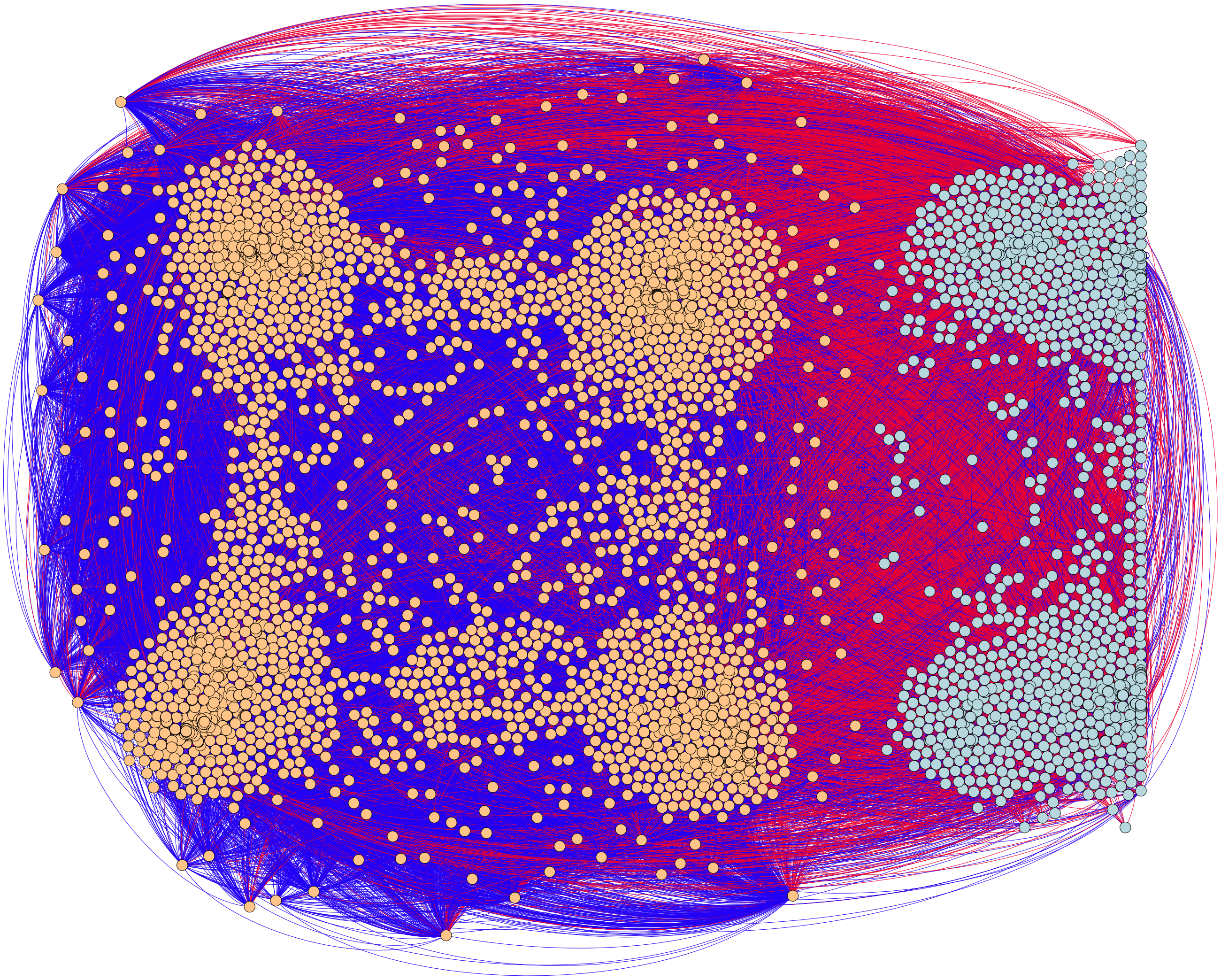

Figure S5. Visualization of Wikipedia network and its optimal partition (high cohesiveness, high divisiveness)

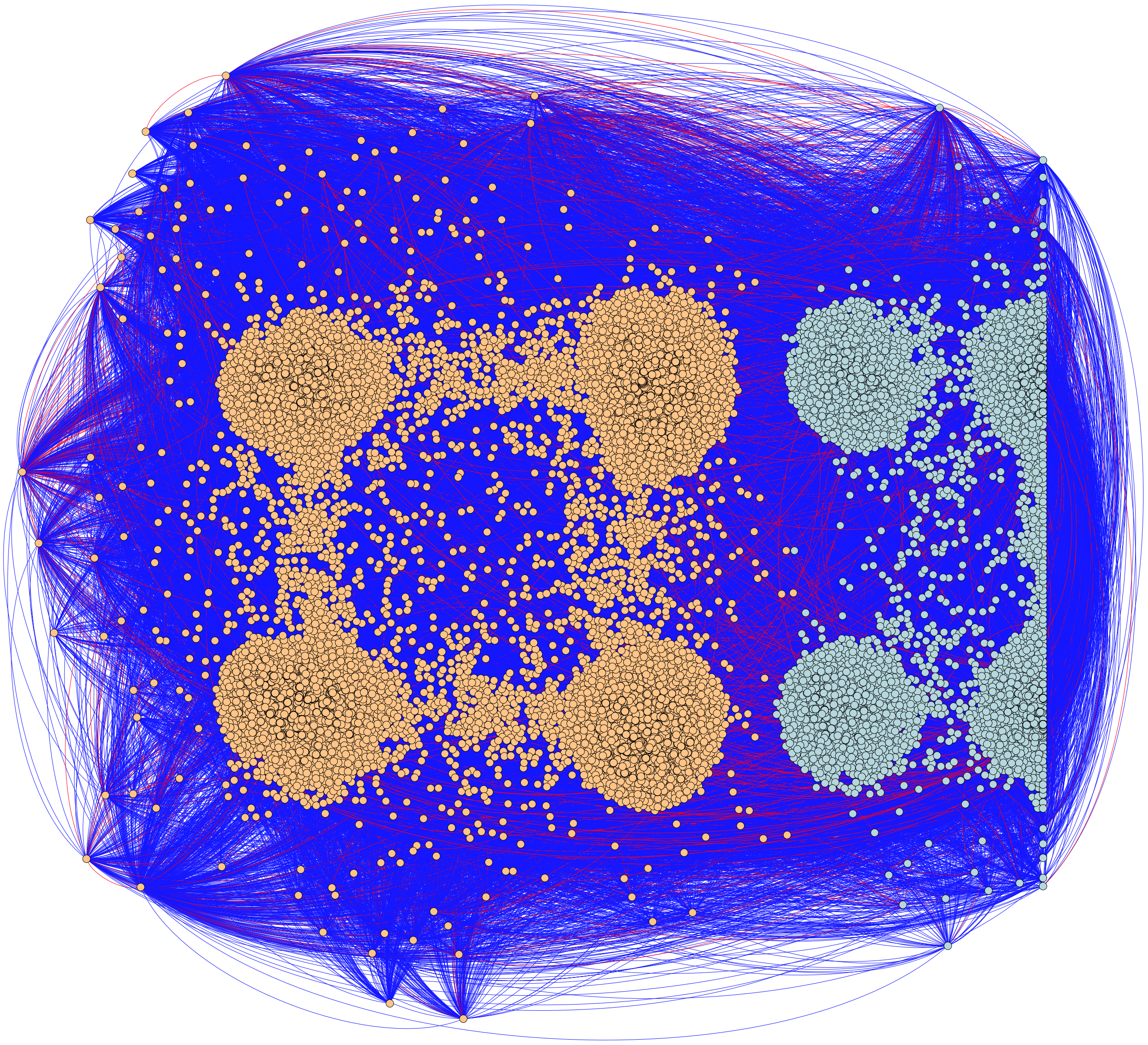

Figure S6. Visualization of Reddit network and its optimal partition (high cohesiveness, low divisiveness)

Movie S1-S3: animated versions of optimal partitions in temporal and multilayer networks

Animated versions of the optimal partitions in temporal networks are available online at https://saref.github.io/SI/ADRD2020/Sampson_partitions.mov for the Sampson affect data (movie S1) and at https://saref.github.io/SI/ADRD2020/Newcomb_partitions.mov for the Newcomb fraternity network (movie S2). The animated visualizations show how the subgroup membership of some of the nodes change over time due to changes in the edges, which in turn alter the optimal partition. The colors used for the nodes represent their optimal group assignment in the first time-frame of the temporal network.

An animated version of the optimal partitions in the multilayer network of philosophers (movie S3) is available online at https://saref.github.io/SI/ADRD2020/Collins_partitions.mp4 The animation shows the two layers of the multilayer network and the flattened network from different orientations.

Dataset S1: directed signed networks from social domain

All networks used in this study are available as edge lists in Comma Separated Value (.csv) format and Geographical Markup Language (.gml) format, and are accessible in a public *FigShare* data repository[33]. These data are distributed under a CC-BY 4.0 license. This means that one can use these data provided that the user provide proper attribution by citing this article and the respective source of original data[17,19,31,32,34–37].

Users of the data in .csv format may notice that for some of the networks, there are values of strength for the edges (values other than $+1,-1$). We have not removed them to keep the dataset more comprehensive. However, in our analysis (and the code which is made publicly available), we load the networks by only considering the signs for the edges and discard any values of strength. Users of the data in .gml format will only see edges with signs of $+1,-1$.

Dataset S2: network-measurements-and-results.csv

All numerical results and additional measurements of the networks used in this study are provided in a .csv file at https://saref.github.io/SI/ADRD2020/network-measurements-and-results.csv. The measurements are described in the column headers, which include the number of nodes n (order), number of edges m (size), number of positive and negative edges m^+,m^-, number of balanced and unbalanced triads, fraction of balanced triads $T(G)$, global clustering coefficient, density, triad census, line index of balance $L(G)$, normalized line index $F(G)$, cohesiveness $C(P^*)$, divisiveness $D(P^*)$, balanced transitive triads by type, and unbalanced transitive triads by type. Each row provides the results and measurements pertaining to a specific network (or a specific time-frame or layer for temporal and multilayer networks) determined by the first column.

Dataset S3: optimal-partitions.csv

The results on optimal partitions (optimal solutions to the optimization model) for all networks are available in a .csv file at https://saref.github.io/SI/ADRD2020/optimal-partitions.csv. Each column refers to a network specified in the first row. Cells in the spreadsheet contain a variable name, index, and its value. For instance, the cell immediately below the header, "Sampson Affect data at T4", contains "x0 : 1" which means that in time-frame T4 of Sampson network, the x variable associated with node 0, takes value 1 in the optimal solution. Up to four types of variables are reported for each network: $x_i, f_{ij}, s_{ij}, t_{ij}$. Variables x_i, f_{ij} are the decision variables of the optimization model which are associated with the nodes and the edges respectively according to their indices. Variable s_{ij} represents the sign of edge (i,j). Variable t_{ij} represents the combination of sign and optimal situation (internal vs. external) for the edge (i,j). There are four combinations for sign and situation denoted by four possible values for t_{ij}: positive internal (with value 3), negative internal (with value 1), positive external (with value -1), and negative external (with value -3).

Supplementary Legends

Fig. S1: Three distinct optimal partitions achievable for college preferences in House A. Direction of arcs are clockwise. Blue arcs are positive and red arcs are negative.
Fig. S2: Two layers of the philosophers network and its flattened view
Fig. S3: Visualization of Bitcoin-Alpha network and its optimal partition (high cohesiveness, high divisiveness)
Fig. S4: Visualization of Bitcoin-OTC network and its optimal partition (high cohesiveness, high divisiveness)
Fig. S5: Visualization of Wikipedia network and its optimal partition (high cohesiveness, high divisiveness)
Fig. S6: Visualization of Reddit network and its optimal partition (high cohesiveness, low divisiveness)
Table S1: Multiple optimal partitions for college preferences in House A and values of cohesiveness and divisiveness
Table S2: Additional numerical results for the networks

References

1. Heider, F. Attitudes and cognitive organization. *The J. Psychol.* **21**, 107–112 (1946).
2. Cartwright, D. & Harary, F. Structural balance: A generalization of Heider's theory. *Psychol. Rev.* **63**, 277–293, DOI: 10.1037/h0046049 (1956).
3. Doreian, P. & Mrvar, A. A partitioning approach to structural balance. *Soc. Networks* **18**, 149–168 (1996).
4. Davis, J. A. & Leinhardt, S. The structure of positive interpersonal relations in small groups. *Sociol. Theor. Prog.* **2**, 218–251 (1967).
5. Harary, F. On the measurement of structural balance. *Behav. Sci.* **4**, 316–323 (1959).
6. Flament, C. Équilibre d'un graphe: quelques résultats algébriques. *Mathématiques et Sci. Humaines* **8**, 5–10 (1970).
7. Zaslavsky, T. Balanced decompositions of a signed graph. *J. Comb. Theory, Ser. B* **43**, 1–13, DOI: 10.1016/0095-8956(87)90026-8 (1987).
8. Aref, S. & Wilson, M. C. Measuring partial balance in signed networks. *J. Complex Networks* **6**, 566–595, DOI: 10.1093/comnet/cnx044 (2018).
9. Aref, S. & Wilson, M. C. Balance and frustration in signed networks. *J. Complex Networks* **7**, 163–189, DOI: 10.1093/comnet/cny015 (2019).
10. Granovetter, M. S. The strength of weak ties. In Leinhardt, S. (ed.) *Social Networks*, 347 – 367, DOI: https://doi.org/10.1016/B978-0-12-442450-0.50025-0 (Academic Press, 1977).
11. Wasserman, S. & Faust, K. *Social Network Analysis : Methods and Applications*. Structural analysis in the social sciences 8 (Cambridge University Press, Cambridge, New York, 1994).
12. Leskovec, J., Huttenlocher, D. & Kleinberg, J. M. Signed networks in social media. In Mynatt, E. D. *et al.* (eds.) *Proceedings of the SIGCHI Conference on Human Factors in Computing Systems*, CHI '10, 1361–1370, DOI: 10.1145/1753326.1753532 (ACM, 2010).
13. Chiang, K.-Y., Hsieh, C.-J., Natarajan, N., Dhillon, I. S. & Tewari, A. Prediction and clustering in signed networks: a local to global perspective. *The J. Mach. Learn. Res.* **15**, 1177–1213 (2014).
14. Rapoport, A. *Mathematical Models in the Social and Behavioral Sciences* (Wiley, New York, 1983).
15. Levi-Strauss, C. *The Elementary Structures of Kinship* (Beacon Press, 1969).
16. Sherwin, R. G. Introduction to the graph theory and structural balance approaches to international relations. Tech. Rep., University of Southern California Los Angeles (1971).
17. Lemann, T. B. & Solomon, R. L. Group characteristics as revealed in sociometric patterns and personality ratings. *Sociometry* **15**, 7–90 (1952).
18. Aref, S. & Neal, Z. Detecting coalitions by optimally partitioning signed networks of political collaboration. *Sci. Reports* **10**, 1–10 (2020).
19. Newcomb, T. M. *The Acquaintance Process* (Aldine Publishing Co, Chicago, IL, USA, 1961).
20. Almaatouq, A., Radaelli, L., Pentland, A. & Shmueli, E. Are you your friends' friend? poor perception of friendship ties limits the ability to promote behavioral change. *PloS One* **11**, e0151588 (2016).
21. Facchetti, G., Iacono, G. & Altafini, C. Computing global structural balance in large-scale signed social networks. *Proc. Natl. Acad. Sci.* **108**, 20953–20958, DOI: 10.1073/pnas.1109521108 (2011).
22. Smith, J. A. & Moody, J. Structural effects of network sampling coverage i: Nodes missing at random. *Soc. Networks* **35**, 652–668 (2013).
23. Maoz, Z., Terris, L. G., Kuperman, R. D. & Talmud, I. What is the enemy of my enemy? Causes and consequences of imbalanced international relations, 1816–2001. *The J. Polit.* **69**, 100–115, DOI: 10.1111/j.1468-2508.2007.00497.x (2007).
24. Estrada, E. & Benzi, M. Walk-based measure of balance in signed networks: Detecting lack of balance in social networks. *Phys. Rev. E* **90**, 1–10 (2014).
25. Lerner, J. Structural balance in signed networks: Separating the probability to interact from the tendency to fight. *Soc. Networks* **45**, 66 – 77, DOI: https://doi.org/10.1016/j.socnet.2015.12.002 (2016).
26. Kleinnijenhuis, J. & De Nooy, W. Adjustment of issue positions based on network strategies in an election campaign: A two-mode network autoregression model with cross-nested random effects. *Soc. Networks* **35**, 168–177 (2013).

27. Lerner, J. & Lomi, A. The free encyclopedia that anyone can dispute: An analysis of the micro-structural dynamics of positive and negative relations in the production of contentious wikipedia articles. *Soc. Networks* **60**, 11–25 (2020).

28. Wang, P., Robins, G., Pattison, P. & Lazega, E. Exponential random graph models for multilevel networks. *Soc. Networks* **35**, 96–115 (2013).

29. Lazega, E. & Snijders, T. A. *Multilevel Network Analysis for the Social Sciences: Theory, Methods and Applications*, vol. 12 (Springer, 2015).

30. Harary, F. On the measurement of structural balance. *Behav. Sci.* **4**, 316–323, DOI: 10.1002/bs.3830040405 (1959).

31. Sampson, S. F. *A Novitiate in a Period of Change: An Experimental and Case Study of Social Relationships* (Cornell University, Ithaca, NY, USA, 1968).

32. Collins, R. *The Sociology of Philosophies* (Harvard University Press, 2009).

33. Aref, S., Dinh, L. & Rezapour, R. Dataset of directed signed networks from social domain. *figshare* http://dx.doi.org/10.6084/m9.figshare.12152628, DOI: 10.6084/m9.figshare.12152628 (2020).

34. Kumar, S., Hamilton, W. L., Leskovec, J. & Jurafsky, D. Community interaction and conflict on the web. In *Proceedings of the 2018 World Wide Web Conference*, 933–943, DOI: 10.1145/3178876.3186141 (ACM, 2018).

35. West, R., Paskov, H. S., Leskovec, J. & Potts, C. Exploiting social network structure for person-to-person sentiment analysis. *Transactions Assoc. for Comput. Linguist.* **2**, 297–310 (2014).

36. Kumar, S., Spezzano, F., Subrahmanian, V. & Faloutsos, C. Edge weight prediction in weighted signed networks. In *2016 IEEE 16th International Conference on Data Mining (ICDM)*, 221–230, DOI: 10.1109/ICDM.2016.0033 (IEEE, 2016).

37. Read, K. E. Cultures of the central highlands, New Guinea. *Southwest. J. Anthropol.* **10**, 1–43 (1954).

38. Johnsen, E. C. Structure and process: Agreement models for friendship formation. *Soc. Networks* **8**, 257–306 (1986).

39. Terzi, E. & Winkler, M. A spectral algorithm for computing social balance. In Frieze, A., Horn, P. & Prałat, P. (eds.) *Proceedings of International Workshop on Algorithms and Models for the Web-Graph*, WAW 2011, 1–13, DOI: 10.1007/978-3-642-21286-4_1 (Springer, 2011).

40. Diesner, J. & Evans, C. S. Little bad concerns: Using sentiment analysis to assess structural balance in communication networks. In *2015 IEEE/ACM International Conference on Advances in Social Networks Analysis and Mining (ASONAM)*, 342–348, DOI: 10.1145/2808797.2809403 (IEEE, 2015).

41. Holland, P. W. & Leinhardt, S. A method for detecting structure in sociometric data. *Am. J. Sociol.* **76**, 492–513 (1970).

42. Bonacich, P. *Introduction to Mathematical Sociology* (Princeton University Press, Princeton, 2012).

43. Zaslavsky, T. A mathematical bibliography of signed and gain graphs and allied areas. *The Electron. J. Comb. Dyn. Surv. Comb. DS8* 1–340, DOI: 10.37236/29 (2012).

44. Aref, S., Mason, A. J. & Wilson, M. C. Computing the line index of balance using integer programming optimisation. In Goldengorin, B. (ed.) *Optimization Problems in Graph Theory*, 65–84, DOI: 10.1007/978-3-319-94830-0_3 (Springer, 2018).

45. Aref, S., Mason, A. J. & Wilson, M. C. A modeling and computational study of the frustration index in signed networks. *Networks* **75**, 95–110, DOI: 10.1002/net.21907 (2020).

46. Stadtfeld, C., Takács, K. & Vörös, A. The emergence and stability of groups in social networks. *Soc. Networks* **60**, 129–145 (2020).

47. Crawford, E. R. & LePine, J. A. A configural theory of team processes: Accounting for the structure of taskwork and teamwork. *Acad. Manag. Rev.* **38**, 32–48 (2013).

48. Cohen, T. R. & Insko, C. A. War and peace: Possible approaches to reducing intergroup conflict. *Perspectives on Psychol. Sci.* **3**, 87–93 (2008).

49. Lau, D. C. & Murnighan, J. K. Demographic diversity and faultlines: The compositional dynamics of organizational groups. *Acad. Manag. Rev.* **23**, 325–340 (1998).

50. Abelson, R. P. & Rosenberg, M. J. Symbolic psycho-logic: A model of attitudinal cognition. *Behav. Sci.* **3**, 1–13, DOI: 10.1002/bs.3830030102 (1958).

51. Iacono, G., Ramezani, F., Soranzo, N. & Altafini, C. Determining the distance to monotonicity of a biological network: a graph-theoretical approach. *IET Syst. Biol.* **4**, 223–235, DOI: 10.1049/iet-syb.2009.0040 (2010).

52. Hüffner, F., Betzler, N. & Niedermeier, R. Separator-based data reduction for signed graph balancing. *J. Comb. Optim.* **20**, 335–360, DOI: 10.1007/s10878-009-9212-2 (2010).

53. Gurobi Optimization Inc. Gurobi optimizer reference manual (2020). Url: gurobi.com/documentation/9.0/refman/index.html date accessed 1 April 2020.

54. Hummon, N. P. & Doreian, P. Some dynamics of social balance processes: bringing Heider back into balance theory. *Soc. Networks* **25**, 17–49 (2003).

55. Ashleigh, M. J. & Stanton, N. A. Trust: Key elements in human supervisory control domains. *Cogn. Technol. & Work.* **3**, 92–100 (2001).

56. Doreian, P., Kapuscinski, R., Krackhardt, D. & Szczypula, J. A brief history of balance through time. *J. Math. Sociol.* **21**, 113–131 (1996).

57. Freeman, L. C. *Research Methods in Social Network Analysis* (Routledge, 2017).

58. Doreian, P. & Krackhardt, D. Pre-transitive balance mechanisms for signed networks. *J. Math. Sociol.* **25**, 43–67 (2001).

59. Belaza, A. M. *et al.* Statistical physics of balance theory. *PLoS one* **12**, e0183696 (2017).

60. Doreian, P. Reflections on studying signed networks. *J. Interdiscip. Methodol. Issues Sci.* **2**, 1–14, DOI: 10.18713/JIMIS-170117-2-1 (2017).

61. Doreian, P. & Mrvar, A. Structural balance and signed international relations. *J. Soc. Struct.* **16**, 1–49 (2015).

62. Srinivasan, A. Local balancing influences global structure in social networks. *Proc. Natl. Acad. Sci.* **108**, 1751–1752 (2011).

63. Monge, P. R. & Contractor, N. S. *Theories of Communication Networks* (Oxford University Press, 2003).

64. Kunegis, J. Konect: the koblenz network collection. In *Proceedings of the 22nd International Conference on World Wide Web*, 1343–1350 (2013).

65. De Domenico, M., Porter, M. A. & Arenas, A. Muxviz: a tool for multilayer analysis and visualization of networks. *J. Complex Networks* **3**, 159–176 (2015).
